# A Hidden Feedback in Signaling Cascades Is Revealed

**DOI:** 10.1371/journal.pcbi.1000041

**Published:** 2008-03-21

**Authors:** Alejandra C. Ventura, Jacques-A. Sepulchre, Sofía D. Merajver

**Affiliations:** 1 Department of Internal Medicine, Division of Hematology and Oncology and Comprehensive Cancer Center, University of Michigan, Ann Arbor, Michigan, United States of America; 2 Institut Non Linéaire de Nice, Université de Nice Sophia-Antipolis, CNRS, Valbonne, France; University of Washington, United States of America

## Abstract

Cycles involving covalent modification of proteins are key components of the intracellular signaling machinery. Each cycle is comprised of two interconvertable forms of a particular protein. A classic signaling pathway is structured by a chain or cascade of basic cycle units in such a way that the activated protein in one cycle promotes the activation of the next protein in the chain, and so on. Starting from a mechanistic kinetic description and using a careful perturbation analysis, we have derived, to our knowledge for the first time, a consistent approximation of the chain with one variable per cycle. The model we derive is distinct from the one that has been in use in the literature for several years, which is a phenomenological extension of the Goldbeter-Koshland biochemical switch. Even though much has been done regarding the mathematical modeling of these systems, our contribution fills a gap between existing models and, in doing so, we have unveiled critical new properties of this type of signaling cascades. A key feature of our new model is that a negative feedback emerges naturally, exerted between each cycle and its predecessor. Due to this negative feedback, the system displays damped temporal oscillations under constant stimulation and, most important, propagates perturbations both forwards and backwards. This last attribute challenges the widespread notion of unidirectionality in signaling cascades. Concrete examples of applications to MAPK cascades are discussed. All these properties are shared by the complete mechanistic description and our simplified model, but not by previously derived phenomenological models of signaling cascades.

## Author Summary

Cellular signaling is carried out by a complex network of interactions. A structure that is found commonly in signaling pathways is a sequence of on-off cycles between two states of the same protein, referred to as a cascade. By analyzing and reducing the basic kinetic equations of this system, we have constructed a new mathematical model of an intracellular signaling cascade. It is widely accepted that information travels both outside-in and inside-out in signaling pathways. Conversely, cascades, even while being main components of those pathways, have been so far understood as structures where signal transmission occurs in a manner analogous to a domino effect: the information flows in only one direction. Adding explicit connections linking a particular level with an upstream location has been the way bidirectional propagation has been explained so far. In other words, up to now, unidirectional cascades would allow bidirectional propagation only when embedded in more complicated circuits. The proposed model shows that a cascade can naturally exhibit bidirectional propagation without invoking extra re-wiring. This result inspires novel interpretations of experimental data; since signaling pathways are usually reconstructed from such data, this outcome could have far-reaching implications in the understanding of cell signaling.

## Introduction

Covalent modification cycles are one of the major intracellular signaling mechanisms, both in prokaryotic and eukaryotic organisms [1]. Complex signaling occurs through networks of signaling pathways made up of chains or cascades of such cycles, in which the activated protein in one cycle promotes the activation of the protein in the next link of the chain. In this way, an input signal injected at one end of the pathway can propagate traveling through its building-blocks to elicit one or more effects at a downstream location.

Examples of covalent modification are methylation-demethylation, activation-inactivation of GTP-binding proteins and, probably the most studied process, phosphorylation-dephosphorylation (PD) [1],[2]. In such cycles, a signaling protein is activated by the addition of a chemical group and inactivated by its removal. This protein is modified in turn by two opposing enzymes, such as a kinase and a phosphatase for PD cycles. In the absence of external stimulation, a cycle exists in a steady state in which the activation and inactivation reactions are balanced. External stimuli that produces a change in the activity of the converting enzymes, shifts the activation state of the target protein, creating a departure from steady state which can propagate through the cascade.

The advantages of these cascades in signal transduction are multiple and the conservation of their basic structure throughout evolution, suggests their usefulness. A reaction cascade may amplify a weak signal, it may accelerate the speed of signaling, can steepen the profile of a graded input as it is propagated, filter out noise in signal reception, introduce time delay, and allow alternative entry points for differential regulation [3]–[5].

Intracellular signaling through cascades of biochemical reactions has been the subject of a great number of studies (e.g., [2],[6] for reviews). Theoretical investigations have been motivated by the increased need for developing an abstract framework to understand the vast amounts of experimental data now available. This whole field of research is further motivated by the hope of characterizing pathways that are deregulated in diseases such as cancer and to define targets to combat these diseases [7].

Since the stimuli a cell receives are varied and complex, cascades do not operate in isolation, but rather the integration of stimuli depends on crosstalk between pathways. Another crucial property of signaling cascades is their ability to integrate information by transmitting the effects downstream and also feedback upstream. In spite of a few decades of intense work on signaling cascades, no models have ever been built that exhibit crosstalk with backwards and forwards transmission of a lateral input from another cascade, except when *ad hoc* feedback is explicitly added to the cascade model. Our model, built from first principles, naturally exhibits these characteristics and therefore inspires novel interpretations of experimental data.

A well studied example of a cascade of activation-inactivation cycles is the cascade of protein kinases. In this case, the basic signaling unit is a PD cycle, whose activating kinase is the phosphorylated protein of the previous cycle. Many proteins contain several phosphorylation sites, allowing for great versatility of regulation. Such is the case, for example, for the mitogen-activated protein kinase (MAPK) cascade, which is widely involved in eukaryotic signal transduction [3], [8]–[10]. For the sake of simplicity, in this article we will mostly consider cascades composed of simple, 2-state activation-inactivation cycles. However, the equations corresponding to the MAPK cascade are also derived and some of their properties compared with those of the simpler cascades. Even though our results are valid in general, for covalent modification cycles, we will employ the nomenclature associated with PD cycles, *i.e*. the converting enzymes will be referred to as kinase/phosphatase.

**Figure 1:**
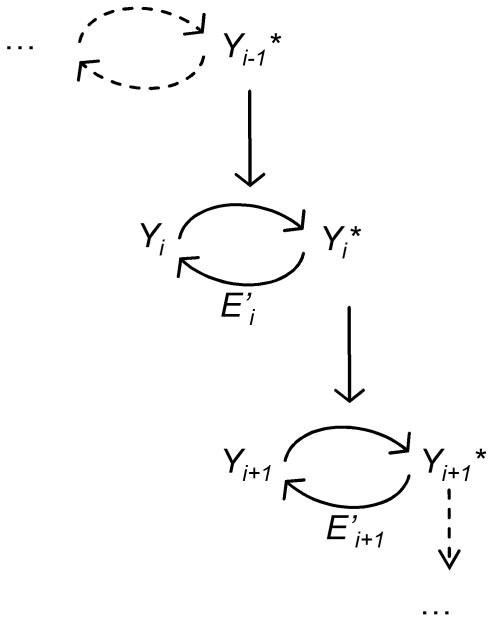
Schematic representation of a cascade of covalent modification cycles. The *i^th^* cycle is composed of two states of the same protein: the inactive and the active states, labeled *Y*_i_ and Y_i_^*^, respectively. In each step, the activation is catalyzed by the activated product of the previous step. The deactivation is performed by another enzyme, *E'_i_*.

The focus of our study is to refine the mathematical modeling of cascades of covalent modification cycles, such us the one depicted in Figure 1. Several mathematical descriptions have been developed to describe such cascades using ordinary differential equations. Typically, those descriptions are built up starting with a model for a single cycle, which is then phenomenologically incorporated into a cascade of cycles. A well known model for describing the single cycle was introduced by the pioneering work of Goldbeter and Koshland (GK) [11]. The GK model considers the concentration of the target protein to be in large excess over those of the converting enzymes, thereby reducing the description to a single equation per cycle. The model obtained in this way was then phenomenologically extended to a cascade of individual GK cycles. Here, by the designation “phenomenological” we mean that, in the cascade, the forward coupling between the GK cycles is chosen as simply as possible, but not strictly deduced from first principles. This phenomenological framework extension of the GK model will be denoted as the *GK-like* model. The GK-like model has been used by several authors to describe the dynamics of signal transduction [9], [12]–[16]. For particular limiting cases, the GK-like model can be simplified further, which results in a model where the inter-converting reactions follow linear rate laws with first-order rate constants. This description was studied in several key papers [17]–[19], and we will refer to it as the *linear rates* model.

The concept of a “cascade” in the study of transduction pathways is appealing because of its modular structure. What is especially appealing is the possibility of defining the cascade state by only one variable per module. As mentioned above, since the building blocks of the GK-like model are the well-studied GK cycles, they involve only one equation per cycle. A different approach however, is to deal with the dynamics of the cascade of Figure 1 by considering the complete set of biochemical reactions and by writing the corresponding equations without any upfront approximations. This was accomplished, for example, for the case of the MAPK cascade [8]. We will refer to this approach as the *mechanistic* model. For the purposes of this paper, we will consider that the mechanistic model represents a complete description of the system under study (event though we recognize that, in reality, it is not a hypothesis-free model).

In this article, starting from the mechanistic description of a cascade composed of an arbitrary number of cycles, we derive a consistent approximation under which the cascade is described with one variable per cycle. It turns out that in this derivation, referred to as a *reduced mechanistic* description, the phenomenological GK-like model is not recovered. At first sight, our new approximation differs slightly from the previously derived description for signaling cascades. However, it involves qualitatively different dynamics from the GK-like model, yet it is in very good agreement with the complete mechanistic description when the approximation conditions are fulfilled.

The main difference between our simplified mechanistic description and the phenomenological one is the appearance of an intrinsic feedback from each unit to the preceding one, caused by the fact that in each cycle there is sequestration of part of the activated protein of the previous step. The new description of the cascade predicts the existence of damped oscillations along the chain, a phenomenon that cannot be observed using the previous phenomenological description. Interestingly, a corollary of our model is that if a particular unit in the middle of the chain receives an input–a common event, given the high degree of crosstalk between signaling pathways–then our reduced mechanistic description predicts that this perturbation is able to travel both forwards and backwards. This “bicistronic” propagation, which may be critical for effective eukaryotic signaling, is not possible within the GK-like description either. Our model provides a suitable framework for future experiments that investigate crosstalk and bicistronic propagation of signals.

## Results

### How to Model a Signaling Cascade

#### A mechanistic description

We consider a cascade of biochemical cycles, as illustrated on Figure 1, in which the two variables *Y_i_* and *Y_i_* ^*^ represent two interconvertible forms of one protein, such as the dephosphorylated and the phosphorylated forms of a kinase; the activated form *Y_i_* ^*^ acts as a catalyst for the next reaction. The cycle between *Y_i_* and *Y_i_* ^*^ constitutes the basic module of a signaling pathway, which comprises *n* such elements. In this cascade, the deactivation occurs by means of a phosphatase denoted by *E′_i_*.

Since processes involved in the control of production of new proteins proceed at a much slower timescale than processes that chemically modify existing proteins, the total quantity *Y_iT_* is considered to be constant in time and the variables [*Y_i_*] and [Y_i_^*^] (the square brackets denote concentration) are then linked by a conservation law. Consequently, only one of the forms, *Y_i_* or *Y_i_* ^*^, will be treated as an independent variable. The first module is activated by an external input signal, that could be, for example, a growth factor or a hormone level. The level of the last protein *Y_n_* ^*^ can be thought of as the “output” of the system.

In an ideal situation, the inter-conversion of the *i^th^* protein can be described by the following reactions:
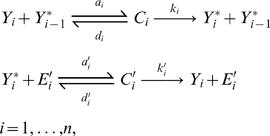
(1)where *C_i_* and *C′_i_* are intermediate enzyme-substrate complexes. Here the conservation law for the protein indexed by *i* is *Y_iT_* = [*Y_i_*]+[Y_i_^*^]+[*C_i_*]+[*C′_i_* ]+[*C_i_*_+1_]. Notice that it comprises the complex concentration [*C_i_*_+1_] formed at the step *i*+1, since Y_i_^*^ activates the (*i*+1)*^th^* cycle. There is also a conservation equation for the reverse enzyme (phosphatase) which can be written as *E′_iT_ = *[*E′_i_*]+[*C′_i_*]. The five variables associated with the module *i*, [*Y_i_*], [Y_i_^*^], [*C_i_*], [*C′_i_* ], [*E′_i_*], are related by two conservation laws, leaving in principle three state variables per cycle. In this setting, the kinetic equations of the cascade can be written using the law of mass action (see Text S1), resulting in what we will call the mechanistic model.

Working in the framework of the mechanistic model offers the advantage that no mathematical approximations are needed (even though, overall, this is obviously not a hypothesis-free model), and this could be the optimal choice for comparing experimental data with numerical simulations of the model. This option was taken, for example, in the context of the MAPK cascade [8].

On the other hand, more complicated models, although in principle more realistic, are also less amenable to developing insights into the transduction pathways. It is appealing then, to find out under which set of hypotheses can the mechanistic model be approximated by a simpler one, for example a model with a single variable per cycle. Arriving to such reduced description is the main contribution of the present paper. Before providing that description, we briefly review some approaches followed in the literature to study signaling cascades by means of only one equation per cycle (see Text S2 for a summary).

#### A model with linear rates

One possible simplification of the chemical reactions in Equation 1 is to neglect the formation of the complexes *C_i_* and *C′_i_*. This can be justified for instance, in the case where the rates *k_i_* and *k′_i_*′s are much larger than the other kinetic constants. Another point of view is to assume that the concentration of each enzyme-substrate complex is very small compared to the total concentration of the reaction partners [17]. Neglecting those complexes in the reactions and then using the law of mass action, one can write the equations for the chain dynamics as follows:
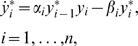
(2)with the definitions *y_i_* = [*Y_i_*]*/Y_iT_* and y_i_^*^ = [Y_i_^*^]*/Y_iT _, Y_iT_* denoting the total available protein. *y*_0_ ^*^ is the normalized input signal and the parameters are *α_i_* = *a_i_Y_T_* and *β*_i_ = *a′_i_E′_i_*. Equation 2 must be complemented by the conservation equation *y_i_*+y_i_^*^ = 1, so here there is indeed a single degree of freedom in the cycle. The nonlinear system in Equation 2 has been dealt with by several authors [17]–[19]. We refer to this model as the linear rates model.

#### An enzymatic model

The linear rates model does not account for the fact that the transformations from *Y_i_* into Y_i_^*^ and from Y_i_^*^ into *Y_i_*, are catalyzed by enzymes. This means that an intermediate enzyme-substrate complex is formed. Therefore, a second class of equations has been considered in the literature to model a covalent modification cycle, taking into account explicitly the enzymatic mechanisms involved. This approach was followed for the first time in the seminal work of Goldbeter and Koshland [11]. Starting with a mechanistic model (but just for a single cycle), one can reduce the description to a single variable by considering that the concentration *Y_T_* is in large excess over those of the converting enzymes. In this way, the enzyme-substrate complexes can be neglected from the conservation equation and they are expressed as a function of the substrates only in the kinetic equations. As usual, this Michaelis-Menten type mechanism is based on a quasi-steady state assumption for the rate of change of the complexes. The resulting equation is then:
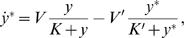
(3)where *y_i_*+y_i_^*^ = 1 and the dimensionless Michaelis-Menten coefficients are defined by *K = (k+d)/(aY_T_)* and *K′* = (*k′+d′*)*/*(*a′Y_T_).* The phenomenological extension of this description for a cascade like the one in Figure 1 is:
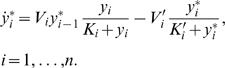
(4)


We will refer to this generalization of the model of Goldbeter and Koshland as the GK-like model. Let us note that in the case where the coefficients *K_i_* and *K′_i_* are much larger than 1, the system in Equation 4 can be approximated by the simpler model of Equation 2 introduced before.

Equation 3 was first derived by Goldbeter and Koshland, to study the so-called property of zero-order ultrasensitivity. This means that when the *K*′s are small (e.g., of the order of 10*^−^* ^2^) the cycle behaves like a switch where the steady state for y^*^ passes abruptly from its lowest to the highest value as a function of the ratio *V/V′*.

The cascade extensions of the Goldbeter-Koshland model have been extensively used in several important articles [9], [12]–[16] covering different contexts, often adding a single negative feedback loop extending from the last unit of the chain to the first one. It has been argued however, that the hypotheses leading to the chain equations (Equation 4) are questionable [3],[20]. Blüthgen et al [3] claim that, in several cases, current experimental data do not support neglecting the enzyme-substrate complexes from the conservation equation. If the affinity of kinase Y_i_^*^ for the protein *Y_i_*_+1_ is high, but its catalytic activity is rather slow, then Y_i_^*^ will remain “sequestered” in the complex *C_i_*_+1_, causing a decrease in available free Y_i_^*^. This phenomenon has been called “sequestration” and it was shown that it can strongly reduce the ultrasensitivity of the chain. If sequestration is important, then the dynamics predicted by the model of Equation 4 is quite different from the one shown by the mechanistic model. Similar arguments are discussed in other work [20]. Moreover, it has been pointed out that the sequestration of part of the activated enzyme of one cycle by the next one has the effect of an “implicit feedback” in the chain [20]. These authors, however, do not carry out a formal analysis of this intuitive statement or of its consequences, as we do in the next section.

The importance of sequestration-based feedback in signaling cascades is thoroughly analyzed in the recent work by Legewie et al [21], where a positive feedback mechanism that emerges from sequestration effects is shown to bring about bistability in the cascade. In that study, sequestration is caused by stable heterodimers formed by the non-phosphorylated protein *Y_i_* and the next substrate *Y_i_*_+1_ in the cascade. Dissociation of this heterodimer is supposed to be induced by the (doubly) phosphorylated protein in cycle *i*+1, entailing a “relief-from-inhibition” positive feedback. In our study however, we point out for the first time a sequestration-based feedback that has been so far overlooked: it exists in the basic model for the MAPK cascade, without invoking any additional mechanism.

### A New Description for Signaling Cascades

In Text S1 we derive in detail the new class of model equations obtained as an approximation of the mechanistic model. The goal of our approach is to reduce the number of variables in the complete system by bringing into play hypothesis that allow us to use the quasi-steady state approximation. Three key dimensionless parameters are defined to facilitate the analysis:
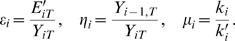
(5)
*ε_i_* and *η_i_* are ratios of total amounts of proteins. *ε_i_* is the ratio of total phosphatase over total targeted protein. *η_i_* is defined as the total targeted protein in one cycle over the corresponding amount in the next cycle in the cascade, or, equivalently, the ratio of total kinase over total targeted protein. The parameter *µ_i_* is the ratio of the kinetic rates of product formation in both the activation and the inactivation reactions (see reactions in Equation 1).

Using a standard singular perturbation analysis, we have found that the state of each biochemical cycle can be described by a single variable defined as x_i_ = y_i_^*^+c_i__+1_, which is the natural slow variable describing the total kinase *i* available at a given time for the phosphorylation in cycle *i*+1. This reduction is only valid if the total phosphatase in the cycle is much lower than the total targeted protein, *i.e.*, in the limit *ε_i_«*1. The other parameters must satisfy *µ_i_ η_i_∼ε_i_*. The dynamics of *x*_i_ is described by the differential equation:
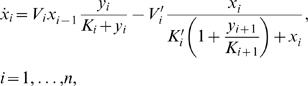
(6)with the following conservation equation from which *y*_i_ has to be extracted:
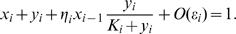
(7)
*x*_0_ *= S* is the normalized input signal and *y_n_*_+1_ = 0. In Equation 6, *V_i_* = (*k′_i _µ_i _η_i_*)*/*(*ε k′*) and *V′_i = _*(*ε_i_ k′_i_*) */*(*ε k′*), where *ε k′* is a typical number representing the set of *ε_i_ k′_i_* (*i* = 1, … ,*n*), e.g. the arithmetical or the geometrical average over this set. In the conservation equation (Equation 7), the notation *O*(*ε_i_*) is just a reminder that this equation is written in the lowest order in *ε_i_*, as is also the case for the differential equation for *x_i_*. In Text S1, we discuss an improvement of this conservation equation which takes into account the first correction in *ε_i_*. Although this extension does not alter the new properties discussed below, its numerical integration is easy and it increases the accuracy of the approximation.

The reduced system given by Equations 6–7 seems to be, in principle, equivalent to the GK-like model given by Equation 4. However, two main features make it significantly different. First, in our novel system, termed the reduced mechanistic model, the conservation equation depends on the variable of the previous cycle. Second and more interesting, the denominator of the negative term in Equation 6 is now a function of the next variable *y_i_*_+1_, in contrast to the GK-like model. This function has the appearance of an effective Michaelis-Menten coefficient *K′_eff,i_ = K′_i_* (1+*y_i_*_+1_ */K_i_*_+1_), which is a typical way to indicate competitive inhibition in enzyme kinetics [22]. In the context of activation-inactivation cycles, a similar type of equation was obtained by Salazar and Höfer in the systematic study of a single cycle taking into account the competition between kinase and phosphatase to bind the same target protein [23]. In that case, an effective Michaelis-Menten coefficient appears also in the negative term of Equation 4, but with the form *K′_eff,i_ = *(1+*y_i_/K_i_*). In our study, however, the competition is induced by the next substrate *y_i_*_+1_, and this precisely describes a negative feedback from cycle *i*+1 on cycle *i*: the higher the level of *x_i+_*_1_, the smaller *y_i_*_+1_ and, therefore, the larger the value of the negative term in Equation 6. This modified denominator reflects the influence of the downstream step on the state variables of one given cycle. It is not a detail of the formalism. It has consequences upon the dynamics and on the properties of the signaling pathway, as it will be demonstrated in the following sections. Moreover, we will see that, since our system arises from a controlled approximation of the mechanistic model, the dynamics of both models can be made comparable.

In the limit *η_i_∼ε_i_«*1, one retrieves the simple conservation law *x*_i_+*y*_i_≈1. However, we note that even in that limit and due to *K′_eff,i_*, our resulting system is not equivalent to the GK-like model. Notice that *η_i_∼ε_i_«*1 is the closest we can be to the hypothesis behind the GK-like model, where it is considered that the concentration of the targeted protein is in large excess over those of the converting enzymes. In our description, the converting enzymes for unit *i* are *E′_iT_* (phosphatase) and *Y_i−_*_1,*T*_ (kinase). Taking the limit *η_i_«*1 together with the fact that the targeted protein of each cycle is the activating protein of the next one, results in increasing protein concentrations as the cascade proceeds. Even though this is not the usual condition in signaling cascades, examples could arise where this limit is suitable. As a possible relevant example, the concentrations reported for the MAPK cascade go from *nM* in the first unit to *µM* in the second and third ones [8].

In addition to the limit *η_i_∼ε_i_«*1, our perturbation scheme encompasses situations where the total protein does not necessarily increase along the cascade. We then allow *η_i_∼*1, for all or for some index *i*, as long as *µ_i_ η_i_∼ε_i_,* which results in the limit *µ_i_∼ε_i_«*1. Since *µ*_i_ = *k_i_/k′_i_*, this limit requires that the phosphatase of that cycle be much more active that the corresponding kinase. In this limit however, the conservation law remains as expressed in Equation 7 and no further simplification can be made. As a result, in this limiting case, the first term in Equation 6 depends on the variable describing the previous step in a different (and more complicated) way compared to Equation 4.

Finally, we notice that our description enables a reduction of the cascade equations with mixed hypothesis concerning the enzymatic reactions. For example, we could have *µ*_1_ *∼ε*_1_ and *η*_1_ *∼*1 for the first cycle, *µ*_2_ *∼*1 and *η_2_∼ε_2_* for the second cycle, etc. Or even *µ_i_∼ε_i_^½^* and *η_i_∼ε_i_^½^* for all or for some index *i*.

In Text S3, we present the extension of the reduced mechanistic model for a cascade involving double-phosphorylation. Notwithstanding that these equations are more complicated than Equations 6–7, the distinguishing feature is maintained: each level in the cascade is subject to influence from the following level which, in the appropriate *x_i_* variable, can be identified as a negative feedback. In the current study we analyze mostly static properties of these more complicated equations and compare them to those of Equations 6–7, while a more exhaustive characterization will be presented in a future article.

### Characterizing the New Model

In this section we report on dynamic and static properties of the new chain equations (Equations 6–7), when studied by numerical simulations, and compare them to those of previous cascade models. We will consider both short (*n* = 3) and long chains (*n* = 10, or 15), respectively. In all the figures we plot y_i_^*^, the level of active protein, obtained from *x_i_* in Equations 6–7 (see Text S4 for a comparison between variables *x_i_* and y_i_^*^). In this section, each parameter in the reduced mechanistic model is considered to be homogeneous throughout the chain, *i.e.*, the parameters do not depend on the index *i* characterizing the position of a particular unit in the chain.

The homogeneity assumption implies that *V_i_*≡*V = µη/ε and V′_i_*≡*V′* = 1. Parameter *S* indicates the level of input stimulation the chain receives. The parameter *K′* is chosen by considering the relation *K′* = *K/µ*. We have performed numerical simulations with other parameter relationships and the properties reported below are not critically dependent upon that choice. The control parameters are, then, *V*, *K*, *µ*, and *η*. Since *V′* = 1, the range of *V* values of interest lies around 1. The initial condition for all the numerical simulations considered is, at t = 0, *x_i_* = 0 (and *y_i_* = 1) for every *i*.

#### Performance of the new approximation

**Figure 2:**
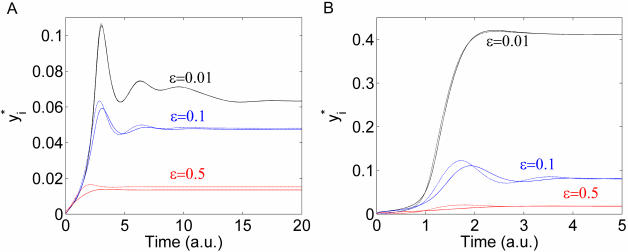
Performance of the new model compared to the mechanistic one. Temporal evolution of the first unit in a chain of 10 units. (A) *η* = *ε* = 0.01, 0.1, 0.5 and *µ* = 1. (B) *µ* = *ε* = 0.01, 0.1, 0.5 and *η* = 1. Other parameters are *K* = 0.01, *K'* = *K/µ*, and *S* = 1. Dashed lines: output of the new model; filled lines: output of the complete mechanistic description.

In Figure 2 we present an initial exploration of the dynamics that the reduced mechanistic model is able to display and how well it approximates the mechanistic model. As an example, a 10-unit chain was considered. The temporal evolution of the variable describing the first unit, *y*_1_ ^*^, is plotted. For each choice of *η* = *ε* (or *µ* = *ε*), the output of the novel reduced mechanistic model is displayed in dashed lines and the predictions of the mechanistic model are depicted in filled lines. The differences between the two descriptions become more noticeable as *ε* increases, as expected. Measuring those differences with the L1-norm, meaning that we compare the curves by computing the difference in the areas under these curves, we find that the reduced model deviates from the complete one less than 0.5%, 4.7%, and 10.6%, for *ε* of 0.01, 0.1 and 0.5, respectively, in Figure 2A. The corresponding values for the percent difference in Figure 2B are 0.9%, 8.3%, and 18%.

We have also computed the errors for less extreme conditions, such as 1) *ε* = 0.1, *η* = *µ* = 0.5; 2) *ε* = 0.1, *η* = 0.5, and *µ* = 1; and 3) *ε* = 0.1, *η* = 1, and *µ* = 0.5 (notice that *ηµ*∼2.5 *ε* for 1) and *ηµ*∼5 *ε* for both 2) and 3)). The respective errors are 4.3%, 3.5%, and 5% (data not shown). The errors in the prediction of the steady states are lower than 0.0001% in all the mentioned cases, indicating the high accuracy of the reduced mechanistic model to study static features of the cascade. This property is due to the conservation equation, Equation 7, taking into account the first correction in *ε_i_* (see Text S1).

#### Appearance of damped temporal oscillations

Interestingly, the temporal evolution of activated protein depicted in Figure 2 exhibits damped oscillations. This feature is displayed by both the mechanistic model and the novel reduced description introduced here. However, such behavior is not attainable within the other available descriptions for signaling cascades models, *i.e*. Equations 2 and 4. Our simplified new description reveals this attribute of the complete (mechanistic) model, that has remained (to our knowledge) hidden until now.

The existence of damped oscillations has been corroborated by a numerical study of stability of the steady state of Equation 6, which is a stable focus. The spectrum of the Jacobian matrix of this system computed at steady-state indeed possesses several eigenvalues with nonzero imaginary parts. The real parts however, are always negative (as observed, e.g., by continuation in *S* parameter) and therefore we cannot obtain sustained oscillations in this chain. A more detailed mathematical study of the spectrum of stability of the chain is beyond the scope of this paper and will be the object of future work. Damped oscillations are not possible without a negative feedback between the cycles [24] and thus reflects the new feature of our model of cascades.

**Figure 3:**
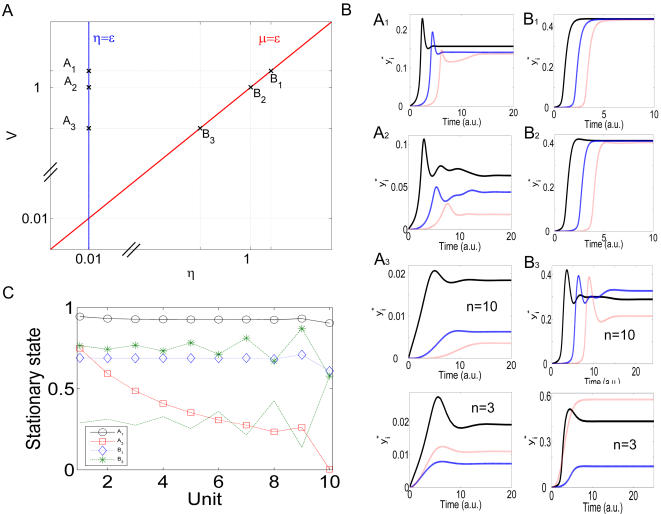
Characterization of the new model's temporal dynamics. (A) Parameter space, *η* on the horizontal axis, *V* = *µη/ε* on the vertical axis (notice that the axes are interrupted). The curves *η* = *ε* and *µ* = *ε* are indicated, and three pairs of values (*η*,*V*) over each of them were selected to show the temporal behavior of the chain. When *η* = *ε*, parameter *V* = *µ* was chosen as 1.2 (A_1_), 1.0 (A_2_), and 0.5 (A_3_), respectively. In the same way, when *µ* = *ε*, parameter *V* = *η* was chosen as 1.2 (B_1_), 1.0 (B_2_), and 0.5 (B_3_), respectively. (B) Temporal dynamics for the selected pairs depicted in (A). *ε* = 0.01, *K* = 0.01, *K'* = *K/µ*, and *S* = 1 for all the panels. The number of units in the chain, *n*, is 10, except for cases A_3_ and B_3_, where both *n* = 10 and *n* = 3 results are shown. In every case, time is plotted in arbitrary units along the horizontal axis and the temporal evolution y_i_^*^ for three of the units in the chain are shown: *y*_1_ ^*^ (black), *y*_4_ ^*^ (blue), and *y*_7_ ^*^ (red). For the case *n* = 3, the same color pattern is used for *y*_1_ ^*^, *y*_2_ ^*^ and *y*_3_ ^*^, respectively. (C) Steady-state achieved by each unit in a chain with *n* = 10, plotted versus the unit number, for the cases A_1_, A_3_, B_1_, and B_3_. For cases A_1_, A_3_, and B_1_, only the results in variable *x_i_* are displayed. Both *x_i_* and y_i_^*^are plotted for parameters B_3_ (dashed-dotted lines with and without stars, respectively.)

Figure 3 contains a representative characterization of the model's temporal dynamics. To simplify the description, we consider two control parameters: *V* = *µη/ε* and *η*, while the other parameters are set as *ε* = 0.01, *K* = 0.01 and *K′* = *K/µ*. The input stimulation is turned on at time 0 from *S* = 0 to *S* = 1. Figure 3A displays the parameter space *η*−*V*. In every panel of Figure 3B, the temporal evolution y_i_^*^ for three of the units in the chain are plotted. In some of the plots, the y_i_^*^′s display damped oscillations before reaching their stationary states. Moving parameter *V* down over each one of the selected curves, *i.e*. from 1.2 to 1 and then to 0.5, enhances the dampening through the chain.

#### Amplified “pathway” oscillations

Another interesting and surprising feature revealed by our new model is that, even though the parameters are homogeneous through the chain, the steady states of variables y_i_^*^ do not always exhibit a monotonous trend with respect to the unit index *i*. This property is evident, for example, in Figure 3B, case B_3_ (*n* = 10) where *y*_4_ ^*^ begins to rise at a later time, but reaches a higher asymptotic value than *y*_1_ ^*^. To study this phenomenon further, in Figure 3C, we plot the steady-state value achieved by each unit in a chain with *n* = 10, versus the unit index, for the cases A_1_, A_3_, B_1_ and B_3_. The positional organization throughout the chain is what we have called “pathway”. Thus, in this sense, B_3_ illustrates “pathway oscillations” that are being amplified along the cascade. The other three examples, when examined in detail, exhibit similar behavior but with less prominent amplification. Notice that for this particular figure we have plotted the variable *x_i_* to better explain the origin of the pathway oscillations in terms of Equations 6–7. The variable y_i_^*^ was included only for the case B_3_ (dashed-dotted line without symbols). A comprehensive explanation of this phenomenon is included in Text S5.

#### Further characterization of the negative feedback between units

**Figure 4:**
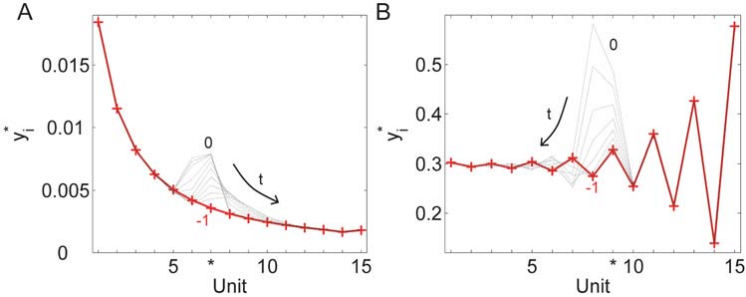
Lateral input is propagated forwards and backwards in the new model. y_i_^*^ is plotted as a function of the index of the unit in the chain, for a chain of 15 units. The status of the chain at t = −1 (in arbitrary units) is indicated with the symbol +, and it corresponds to the steady-state situation. At t = 0, the indicated unit (see asterisk on the horizontal axis) receives a perturbation Δ*x*, which is then propagated to other units. Times 1 to 10 are plotted in dotted lines. The parameters are (A) *η* = *ε* = 0.01 and *µ* = 0.5, (B) *µ = ε* = 0.01 and *η* = 0.5. The remaining parameters are *K* = 0.01, *K'* = *K/µ*, and *S* = 1 in both (A) and (B).

Let us consider a chain that is in equilibrium, *i.e.*, a cascade in which every unit has achieved steady-state. We then perturb a single variable *x_i_*, as indicated in Figure 4. The nature of our new description, that couples each unit with both the previous and the following one, makes it possible to transduce the localized perturbation in both directions, forward and backward. Figure 4A and 4B, which correspond to parameters A_3_ and B_3_ respectively, illustrate cases where the propagation occurs mainly forward or mainly backward. However, propagation in both directions simultaneously is also possible. We observe that Figure 4B, where the propagation occurs mainly backwards, has stronger feedback than Figure 4A since *K/K′* has a value of 10 for B and 2 for A.

#### Stimulus-response curves

**Figure 5:**
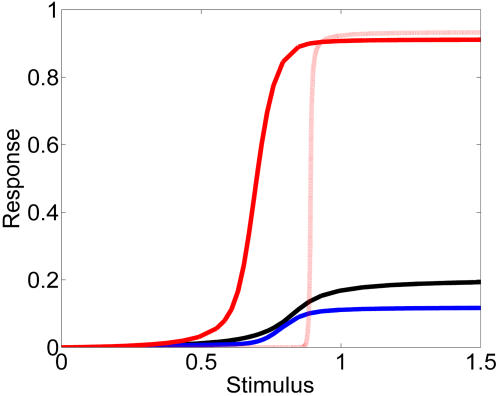
Stimulus-response curves. Stimulus-response curves corresponding to parameters A_1_ in Figure 3, for a chain with three units. The stimulus strength is the value of *S* and the response is y_i_^*^. Variables associated with units 1, 2, and 3 are plotted in filled black, blue, and red lines, respectively. The stimulus-response curve *y*_3_ ^*^ for the GK-like model is superimposed in red dotted lines.

We now study the time-independent features of our model (Equations 6–7), and compare them with those of the system described by Equation 4 for the same set of parameters. The stimulus-response curve is defined, as customary, by the steady-state values of the variables as a function of the input stimulus *S* (recall that in Equation 6, *x*_0_ = *S*, which is assumed to be constant in time). Figure 5 shows the stimulus-response curves obtained with Equation 6 (filled lines) for condition A_1_ from Figure 3A, except for the dotted line that was calculated with the GK-like model (Equation 4). We observe that with the parameters so assigned, the computations performed with the reduced model deviate by less than 0.0001% from the (non approximated) mechanistic model. The results were obtained with a chain of three units, as an example.

In Figure 5, the variables y_i_^*^ display sigmoidal responses. The low level of activation for units 1 and 2 is due to the fact that the proteins are indeed partially sequestered in the enzyme-substrate complexes. However, *y*_3_ ^*^, having no possible sequestration, achieves then a much higher steady state than the other units, and this steady-state is comparable to the one predicted by the GK-like model. In contrast, the predictions of the GK-like model for units 1 and 2 diverge significantly from those of our new model (data not shown). *y*_3_ ^*^ in the GK-like model responds in a steep manner due to the characteristic ultrasensitivity of this model. The same variable computed with our equations responds in a less steep way, this disparity could be interpreted, as suggested in the work of Blüthgen et. al. [3], by the fact that our approximated model, Equations 6–7, which gives the same output as the full system, takes into account the sequestration phenomenon. These ideas are expanded in the following section.

### The New Model Applied to a Known Pathway: Comparison with Experimental Data

In this section we apply the reduced mechanistic model to a well-known signaling pathway, the mitogen-activated protein kinase (MAPK) one [3], [8]–[10],[25],[26]. We first base our description on a particular published set of parameters for this pathway [8]. Importantly, the results obtained are not qualitatively modified by variations of the selected values in the ranges suggested in the literature [8]. Moreover, they are not modified by choosing different sets of parameters [9],[25],[26], as described in the Text S6.

It is well know that the MAPK cascade consists of three levels, the second and the third ones involving a double-phosphorylation mechanism. In this section we consider both the MAPK cascade and a simpler case, a 3-unit chain where each unit is a 2-state cycle.

Starting with the published set of parameters (see [8] and also [10], for a summary), we have computed the parameters involved in the reduced mechanistic description and listed them in Table 1. As described in Text S6, there are some extra parameters for the case involving double-phosphorylation, that are designated *ν*, K^*^, and *K″* and take the values of 1, 0.25, and 0.25, respectively.

**Table 1:** Parameters involved in the reduced mechanistic description corresponding to the set of parameters published in [8] for the MAPK cascade.

Unit	*ε*	*η*	*µ*	*K*	*K'*
1	0.1	0.1	1	100	100
2	0.00025	0.0025	1	0.25	0.25
3	0.1	1	1	0.25	0.25

According to Table 1, the conditions under which the reduced model is valid are only partially satisfied, *ηµ*∼*ε* for the first unit but *ηµ*∼10 *ε* for the second and third ones. Even for these conditions and since the focus of this section is in steady states, the reduced mechanistic model provides a description that is in excellent agreement with the complete mechanistic one.

**Figure 6:**
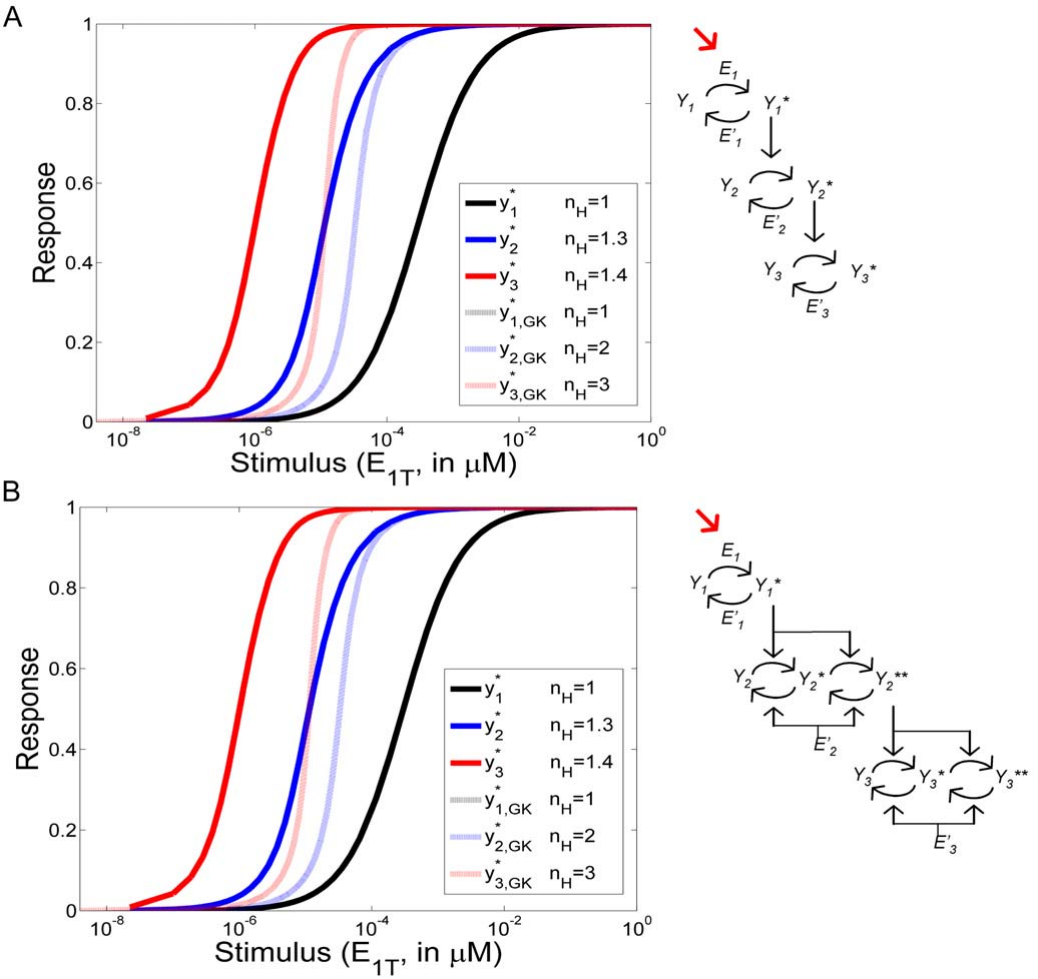
Stimulus-response curves for a 3-unit chain. Stimulus-response curves for a 3-unit chain involving only single phosphorylation (A) or with double phosphorylation in units 2 and 3, representing the MAPK cascade (B). The parameters are those indicated in Table 1. The responses were obtained by both the mechanistic and the reduced mechanistic descriptions, which are in perfect agreement. The input stimulus is given by *E*_1*T*_ , the total amount of kinase for the first unit. *y*_1_ ^*^, *y*_2_ ^*^, and *y*_3_ ^*^ are plotted with black, blue, and red filled lines, respectively. GK-like model predictions are also included (dotted lines). The Hill coefficients characterizing each curve are listed in the legend.

In Figure 6 we plot the normalized stimulus-response curves for a 3-unit chain, either with single-phosphorylation in all the units (A) or with single-phosphorylation in unit 1 and double-phosphorylation in units 2 and 3 (B), *i.e.*, the case corresponding to the MAPK cascade. Both cases are characterized by the parameters in Table 1. The input stimulus was taken to be the concentration of *E*_1*T*_ , the total amount of kinase for the first unit in the cascade (corresponding to MAPK kinase kinase in B). *E*_1*T*_, related to the parameter *S* we have used as input in the previous section, was varied over a wide range. The outcomes were obtained by both the complete mechanistic and the reduced mechanistic models and the results are indistinguishable for the scales of the figure (black, blue, and red filled lines for *y*_1_ ^*^, y_2_ ^*^, and y_3_ ^*^, respectively). For completeness, we are also including the corresponding outcomes obtained by the GK-like model (dotted lines).

In order to compare the steepness in the responses, we have computed the apparent Hill coefficient *n_H_* ([8]) for each curve, as indicated in the legend. As expected, *n_H_* increases through the chain. Moreover, *n_H_* is also considerably reduced when comparing GK-like model's predictions with the predictions of both mechanistic and reduced mechanistic models (which are, as already mentioned, undistinguishable). As explained in the section dealing with stimulus-response curves, these differences could be due to the fact that both the mechanistic and the reduced mechanistic descriptions take into account “sequestration” in the enzymatic reactions [3].

We have mentioned that the good agreement between the mechanistic and reduced mechanistic descriptions regarding the prediction of steady states is due to the conservation law, Equation 7, taking into account the first correction in *ε_i_* (see Text S1). If that correction is not considered, differences could appear in the steady states predicted by the mechanistic model and the reduced mechanistic one. However, and for the parameters in Figure 6, the predicted values of *n_H_* are not modified by removing the *ε_i_* correction in the conservation law or, even by, removing the *η_i_* correction as well (*i.e.*, using a conservation law of the form *x_i_*+*y_i_* = 1). These results strongly indicate the robustness of the new equations regarding the “ultrasensitivity” characteristics of the cascade.

In Figure 6B, the mechanistic and reduced mechanistic models' outcomes and corresponding Hill coefficients recover published results [8]. Comparing figures A and B, we also confirm that the chain involving double-phosphorylation responds in a steeper manner than the one with only single-phosphorylation, as expected from previous work [27].

**Figure 7:**
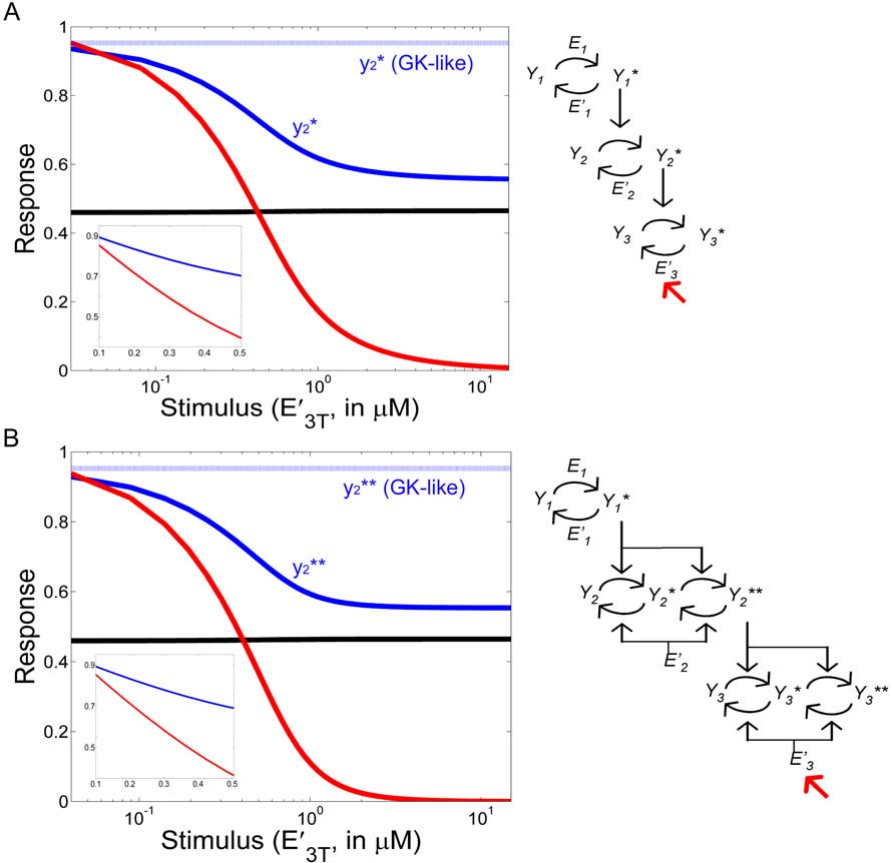
“Reverse” stimulus-response curves. “Reverse” stimulus-response curves for a 3-unit chain involving only single phosphorylation (A) or with double phosphorylation in units 2 and 3, representing the MAPK cascade (B). The parameters are those indicated in Table 1. The responses were obtained by both the mechanistic and the reduced mechanistic descriptions, which are in perfect agreement. The input stimulus is given by *E'*_3*T*_, which is the total amount of phosphatase for the last unit. *y*_1_ ^*^, *y*_2_ ^*^, and *y*_3_ ^*^ are plotted with black, blue, and red filled lines, respectively. GK-like model predictions are also included (dotted lines). Insets show details of the figures.

In Figure 7 we show the outcome of stimulating the 3-unit chain as indicated in the schemes close to each panel: the input stimulus to the cascade was taken to be the concentration of *E′*_3*T*_, the total amount of phosphatase for the last unit in the cascade (corresponding to MAPK in B). *E′*_3*T*_ was varied over its suggested range of variation [8]. Increasing the amount of phosphatase produces a decrease in the response curve *y*_3_ ^*^ (red filled line), as expected. Interestingly, our new reduced model (Equations 6–7), as well as the complete mechanistic description, predict that this perturbation on the third level of the chain is propagated backwards: the variation in *y*_2_ ^*^ is actually a decrease due to a higher sequestration of free *y*_2_ ^*^ by the next step in the chain caused, in turn, by the increased demand of *y*_3_. This result is exhibited by both cascades in Figure 7 (the one involving only single-phosphorylation and the one with double-phosphorylation in units 2 and 3) and we call it “reverse” stimulus-response curves. As stated before, this result is obtained with both the mechanistic and the reduced mechanistic descriptions, with realistic parameters associated with a well studied signaling pathway, such as MAPK.

The insets in both figures indicate that is not necessary to vary parameter *E′*_3*T*_ over a wide range to observe this property, rather it is clearly seen by changing it only by a factor 5 around its suggested concentration (0.12 *µM*), where a 20% variation in *y*_2_ ^*^ is observed, a value that is high enough to be detected experimentally (meaning that it is most likely not contained within the error of the experiment). Due to the parameters characterizing this particular pathway, the effect is not propagated to *y*_1_ ^*^ (black filled line), but this fact does not have to be generalized (see Text S6). The dotted horizontal lines in Figures 7A and 7B are the GK-like prediction for the response curve *y*_2_ ^*^: within that phenomenological description, a particular level in the cascade is not at all influenced by what happens in a downstream unit. However, this well known property of unidirectional influence in a signaling chain, which is embodied by the appellation of “cascade”, is shown here not to be guaranteed in general signaling cascades.

In Text S6 we extend the results in this section concerning “reverse” stimulus-response curves, for different sets of published parameters on the MAPK cascade.

### Modular Response Analysis of the Cascade

A modular response analysis (MRA) [28] was applied to determine the network architecture of the cascade in the context of the new model equations (Equations 6-7). MRA has recently been proposed as a tool to characterize the interactions between “modules” in a cellular regulatory network, having the advantage of allowing direct experimental implementation.

As a matter of fact, the negative sign of the Jacobian element *∂x* ˙*_i_/∂x_i_*_+1_ indicates that the (*i*+1)*^th^* level of the cascade exerts a negative effect in what concerns variable *x_i_*. This effect (what we have called “negative feedback”) is intrinsic, as opposed to “explicit” negative feedback which is sometimes considered in models of signaling pathways [9],[12],[13]. MRA is, then, an appropriate approach to test this bidirectional structure and to estimate the relative strength of the backward interaction, as compared with the forward coupling in a signaling cascade.

As a result of applying MRA, a matrix of local response coefficients **r** is obtained. An element *r_ij_* in this matrix describes how the state of the variable associated with module *j* directly influences the state of the variable associated with module *i*. More precisely, a response coefficient *r_ij_* lower/greater than 1 means that a relative change in module *j* is attenuated/amplified in module *i* by a factor *r_ij_* (*i.e.*, Δ*x_i_/x_i_* = *r_ij_* Δ*x_j_/x_j_*). A zero response coefficient indicates no direct effect between the involved modules, whereas a negative response coefficient means inhibition. In this way, the matrix **r** provides an interaction map to characterize the type and strength of the interactions between the modules in a cellular regulatory network.

Indeed, if the rate of change of variable *x_i_* is denoted by the function *f_i_*, it easily can be shown that:
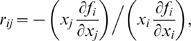
(8)meaning that *r_ij_* corresponds to a scaled version of the Jacobian matrix *∂f_i_/∂x_j_* (evaluated in the steady state). Moreover, it was proven that the local response matrix **r** can be obtained from another matrix named global response matrix, **R** *_p_*, that has the advantage of being accessible experimentally [28]. For example, the element (*i*, *j*) of this matrix can be obtained by perturbing a parameter *p_j_* affecting only module *j* and computing the relative changes induced on the steady state of *x_i_*, namely (Δ*x_i_/x_i_)/*Δ*p_j_.* For more details about the broad scope of the method, we refer the reader to the cited reference and references therein.

**Figure 8:**
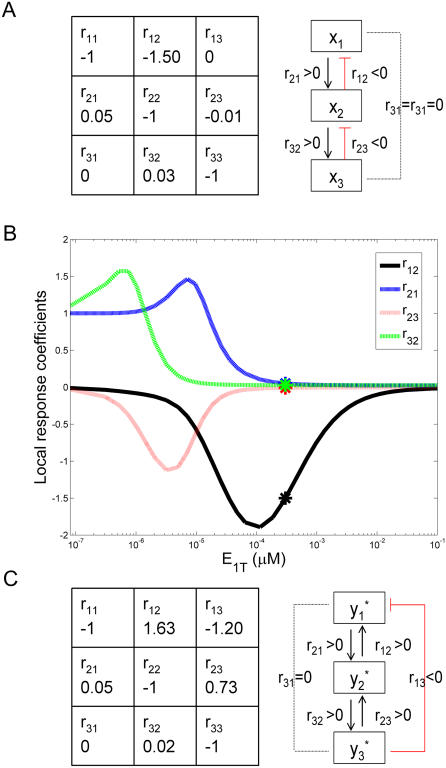
Modular response analysis (MRA). Modular response analysis (MRA) applied to the new model for signaling cascades. MRA was applied to a 3-unit cascade involving only single phosphorylation and characterized by the parameters in Table 1. (A) Interaction map and reconstructed network topology regarding variables *x_i_*. (B) Local response coefficients (regarding *x_i_*) versus parameter *E*_1*T*_. Black, blue, red, and green for *r*_12_, *r*_21_, *r*_23_, and *r*_32_, respectively. The asterisks over each curve indicate the values of the matrix in (A), corresponding to *E*_1*T*_ = 3×10*^−^* ^4^ *µM*. (C) Interaction map and reconstructed network topology regarding variables y_i_^*^.

Using notations and concepts from the literature [28], we apply the MRA method to a 3-unit cascade involving only single-phosphorylation and characterized by the parameters in Table 1 [8]. There are three modules in this network as described by Equations 6-7, each of them corresponding to the three successive levels in the cascade and characterized by a single variable *x_i_*. Figure 8A contains the matrix of local response coefficients **r**. This matrix was obtained both by direct computation of the scaled Jacobian matrix (Equation 8) and by simulating experimental perturbations to the cascade, then computing the global response matrix, **R** *_p_*, and finally obtaining **r**, as described previously [28] (details of second calculation not shown). Using MRA, the “theoretical” and “experimental” outputs were in perfect agreement and the results are displayed in Figure 8A.

The structure of matrix **r** is tridiagonal, meaning that the first level in the cascade does not directly influence the third one (*r*_31_ = 0), and viceversa (*r*_13_ = 0). Coefficients *r*_21_ and *r*_32_ are positive, representing the positive effect of each level in the cascade to the subsequent one. Interestingly, *r*_12_ and *r*_23_ are both negative, indicating an inhibitory effect from unit (*i*+1) to unit *i*. The resulting connections between the units in the cascade are summarized in the scheme in Figure 8A.

To understand these results in more depth, we have studied how the coefficients in the matrix in Figure 8A depend on the parameters characterizing the cascade. For example, Figure 8B shows coefficients *r*_21_, *r*_32_, *r*_12_, and *r*_23_ versus the parameter *E*_1*T*_. *r*_31_ and *r*_13_ are zero (data not shown), *r*_21_ and *r*_32_ are positive, and *r*_12_ and *r*_23_ are negative, throughout the range where *E*_1*T*_ was varied. Depending on the value of *E*_1*T*_, each of the nonzero *r_ij_* could be less or greater than 1 and the relative strength of the backward and forward couplings for a given pair of modules, e.g. |*r*_12_ */r*_21_|, could exhibit large variations. Similar curves have been reported in the literature for signaling cascades [29], but lacking the information about *r*_12_ and *r*_23_, which have always been considered to be zero in previous papers.

Studies like the one in Figure 8B help us understand and also predict the degree of backwards coupling as a function of the parameters in the model. One utility of this work is as a starting point of a more systematic study on how to enhance or attenuate that coupling in the cascade, the subject of our ongoing work.

Interestingly, the interaction map characterizing the connectivities between variables *x_i_* (matrix **r**(*x_i_*) in Figure 8A) shows strong differences when compared to the one computed for the “free” enzyme variables y_i_^*^ (matrix **r**(y_i_^*^) in Figure 8C. Although an explicit set of differential equations is not written for the variables y_i_^*^, the matrix **r**(y_i_^*^) can be calculated using the “experimental”' method described in the literature [28]. The result in Figure 8C is the average of four outputs and the corresponding error (standard error of the mean) is lower than 4%. As indicated in the reconstructed topology close to the matrix, *r*_12_ and *r*_23_ are now positive (as are *r*_21_ and *r*_32_), *r*_31_ is zero, and *r*_13_ is negative, indicating an inhibitory coupling from variable *y*_3_ ^*^ to variable *y*_1_ ^*^. The matrix **r**(y_i_^*^) is consistent with the results in Figure 7A (and also those in Text S6): in other words, the response in *y*_2_ ^*^ goes in the same direction as the one in *y*_3_ ^*^ (whereas plotting variables *x_i_* indicates a decrease in *x*_3_ and an increase in *x*_2_, data not shown).

Experimental data concerning the application of MRA to the MAPK cascade are now available in the literature [30], showing non zero *r*_21_ and *r*_32_ coefficients (and also non zero *r*_31_ and *r*_13_ coefficients). The interpretation of non zero *r*_31_ and *r*_13_ was proposed in terms of the usual “explicit” positive or negative feedbacks which are sometimes considered in models of signaling pathways [9],[12],[13]. From this perspective, the explanation for the non zero *r*_12_ and *r*_23_ coefficients was, at least regarding *r*_23_ and based on experimental evidence, that not only is *y*_2_ ^*^ able to phosphorylate *y*_3_, but *y*_3_ ^*^can phosphorylate *y*_2_ as well [30],[31]. Our results however, suggest that the non vanishing backward coefficients (*r*_12_, *r*_13_, *r*_23_) can be accounted for, at least partly, by the natural “implicit” feedback which can exist in a signaling cascade. A quantitative correlation between these recent experimental results and our predictions is not possible at this time. In the published experiments, the MAPK cascade is not isolated but embedded in the complex cellular machinery; therefore, the measured connectivities could involve proteins external to the cascade itself and it would be premature to establish the connection with our simplified model. Nevertheless, the work in [30] suggests a direction for the type of experiments that could validate our results.

## Discussion

The main contribution of this work is to propose a new one-variable per cycle model for signaling cascades of covalent modification cycles, consistent with a mechanistic complete description. Our model reveals new and biologically relevant properties of such cascades. These properties are characterized completely for the case of single-phosphorylated cascades. Furthermore, single and doubly-phosphorylated cases are compared regarding their stimulus-response curves, while a more exhaustive characterization of the scheme involving double phosphorylation will be presented in a future article.

The scheme in Figure 1, which has been employed by many groups, is suggestive of the concept of a “cascade”. From a systemic point of view, a cascade is a system composed of units, the output of which is successively an input to the next unit. Based on this structure, powerful concepts from control theory can be applied successfully to the study of signaling cascades [14]. Although these concepts have proven its utility in many contexts, this kind of schematic representation implicitly conveys the idea that a signaling cascade is only a feed-forward chain in which signal transmission is analogous to a domino effect [32],[33]. Our study sheds a different light on this system, showing that this schematic representation can be misleading, since it turns out that each unit is actually coupled not only to the following one but also to the previous one, and interesting dynamics can arise from these interactions.

Our initial motivation for developing a new one-variable description of signaling cascades, was the following observation. The main assumption underlying the GK description of a single cycle is that the concentration of the target protein is in large excess compared to those of the converting enzymes. Holding the same assumption over a cascade of units would mean that the target proteins are in higher and higher concentration as the cascade progresses, since they act as the transforming enzyme for the following cycle. To our knowledge, this important issue has not been remarked upon in the literature, except for a brief comment in the work of Millat et. al. ([20], page 11).

In order to get more insight into this point, we have sought special limiting cases for which the mechanistic and the GK-like model are in good agreement. However, it turns out that the dynamics of the signaling cascade described by the mechanistic and the GK-like models cannot be compared consistently. The fundamental reason for this mismatch is that a careful perturbation analysis applied to the mechanistic model provides a different set of equations.

We note that in search for an adequate set of hypothesis leading from the mechanistic equations to the model given by Equations 4, we have studied an alternative scheme in which the modified protein *Y_i_* ^*^ is not directly the kinase of the next reaction. Instead, we studied the case where *Y_i_* ^*^ activates that kinase. This scheme was suggested by the work of Goldbeter [12]. The resulting equation (see Text S7) is fundamentally different from the GK-like model. In reality, no set of assumptions can give rise to the GK-like model as a limiting case of our model.

Our mathematical method relies on the standard quasi-steady state assumption (QSSA), which can be applied under well defined conditions to elicit a clear separation between the slow and fast dynamics of the mechanistic model. Under this standard QSSA framework, our analysis shows that a good slow variable for which evolution equations can be written is the sum of the free activated enzyme which is available in the *i^th^* cycle plus the amount of this protein which is captured by the next inter-converting cycle. The idea of working with a mixed variable *x_i_* can be further generalized by considering the “total” variable corresponding to the total amount of activated enzyme found not only as free molecules or bound to the next substrate, but also complexed with the reverse enzyme *E′_i_*. In fact, this choice is the key ingredient of the method called the “total” quasi-steady state approximation (tQSSA) which has been proved to be a simple but most efficient extension of the standard QSSA [34]. The application of this extended framework to the description of the signaling cascade of Figure 1 is concerned with our current research. In the same context, other authors have recently applied the tQSSA method to the study of small networks of GK cycles [35]. These systems do not form cascades, but involve a more complicated coupling between the units. Nevertheless, their results show that indeed the tQSSA method is successful in obtaining a reduced set of equations, with one variable per cycle, which faithfully reproduces the dynamics of the network for a large range of system parameters.

Even in the less extended QSSA framework, the conditions under which the model is valid are made clear. Under such conditions, our new model is indeed in perfect agreement with the complete mechanistic model (Figure 2). Those conditions are expressed in terms of three key parameters (Equation 5) we have defined to simplify the study. Even though the phenomenological equations, Equation 4, are appealing because of their simpler form and modular nature, we could not find any set of assumptions that would enable us to recover those descriptions. Our simplified model reveals properties of signaling cascades that were either hidden by the complex structure of the complete mechanistic model or lost in the simplified phenomenological descriptions.

It was stated that the reduced mechanistic model is valid whenever these two conditions are satisfied: *ε_i_«*1 and *µ_i_ η_i_∼ε_i_*. The study of the performance of the new approximation (Figure 2 and the corresponding computed errors) makes it clear that even when those conditions are satisfied only moderately, the new model is still robust in approximating the complete description. As an example, we have computed a 5% error for *ε* = 0.1, *η* = 1, and *µ* = 0.5 (meaning *µη∼*5*ε*). Moreover, we have observed that the steady state predictions of the reduced model are highly accurate. Therefore the properties of signaling cascades we are unveiling thanks to the new reduced model, are not restricted by a tight relationship between concentrations and reaction rates hard to achieve in *in vivo* or *in vitro* conditions.

All the novel properties of a signaling cascade reported in this paper are linked, as previously mentioned, to the negative feedback from each unit to the previous one. This backward negative feedback can produce damped temporal oscillations in the chain, or it can create amplified “pathway” oscillations in the steady states of the cascade. Interestingly, it can also transduce a signal both forward and backwards. Given the multi-branched complex nature of many signal transduction pathways, this finding could have wide implications and can help focus further experimental investigation.

It has recently been reported that the 3-level MAPK cascade has autonomous oscillations without any kind of added explicit feedback [36]. Following a systematic numerical exploration of the corresponding mechanistic model [8], the authors provide a qualitative description of the mechanism responsible for these sustained oscillations. Their explanation strongly suggests the necessity of a bistable behavior at the second or third levels of the cascade, thus requiring double-phosphorylation at these stages [37]. Consistent with their findings, we have observed only damped oscillations in the dynamics of the single-phosphorylated cascade (Equations 6–7), which has been the main focus of the present work. Interestingly, preliminary numerical simulations of our reduced doubly-phosphorylated cascade model (Text S3), indicates that these autonomous oscillations are recovered in the simplified description.

The stimulus-response curves of the new model were also investigated (Figure 5). They have the usual sigmoidal shape characteristic of ultrasensitive responses; however, they exhibit lower steepness when compared with the output of the GK-like model. This result corroborates the conclusions stated in the work of Blüthgen et al. [3], where an analysis of the effect of sequestration was conducted. This effect is partially mitigated by double-phosphorylation (Figure 6), as expected from the literature [27].

To further characterize the new model within realistic conditions, we have studied it subject to different sets of published parameters corresponding to a well-known signaling pathway, such as the MAPK one (Figures 6 and 7, and Text S6). We have found that the ability of the model to transduce a signal both forward and backwards is widespread and that the effect is of enough magnitude to allow experimental verification.

Finally, we have applied a modular response analysis to determine the network architecture of the cascade described by the new model equations (Figure 8). This well-known approach enables not only to test the bidirectional structure of the cascade, but also to estimate the relative strength of the backward interaction.

In summary, our findings do not at all weaken the importance of previous models like the GK-like models and those with linear rates. To the contrary, the results of our model provide a different approach to deal with a simple one-variable per cycle model to describe signaling cascades. We hope that our contribution will help in the understanding of existing models for signaling cascades, will improve the description of available data, and will inspire both theoretical and experimental investigation.

## Methods

All the ODEs were integrated in MATLAB 7 (Mathworks, Natick, MA). The stimulus-response curves were obtained using MATCONT, a MATLAB package for numerical bifurcation analysis of ODEs. The symbolic calculations were done using the Symbolic Math Toolbox in MATLAB.

## Supporting Information

Text S1 Perturbation analysis of the mechanistic model.
(0.06 MB PDF)

Text S2 Available models for signaling cascades using one variable per unit.
(0.06 MB PDF)

Text S3 Cascades involving double phosphorylation.
(0.23 MB PDF)

Text S4 Comparison variables *x_i_* and y_i_^*^.
(0.49 MB PDF)

Text S5 About the amplified “pathway” oscillations.
(0.05 MB PDF)

Text S6 More on “reverse” stimulus-response curves.
(0.53 MB PDF)

Text S7 A variation of the model with an intermediate step in the kinase activation.
(0.21 MB PDF)
