# Supplementary material for: A Hidden Feedback in Signaling Cascades Is Revealed
Source: PLoS Comput Biol. 2008 Mar 21;4(3):e1000041. doi: 10.1371/journal.pcbi.1000041 (PMC2265423; doi:10.1371/journal.pcbi.1000041)
Supplement: Text S1 — Perturbation analysis of the mechanistic model. (0.06 MB PDF) [file pcbi.1000041.s001.pdf]

Text S1
Perturbation analysis of the mechanistic model

We show how our new model equations (Eqs.(6)-(7)) are obtained as an approximation of the mechanistic model.

First, the kinetic equations describing the cascade are written using the law of mass action (the resulting system is what we call the mechanistic model). According to the scheme in Eqs. (1), the dynamics of the ith cycle in a cascade of n cycles is governed by the conservation equations $Y_{iT} = [Y_i] + [Y_i^*] + [C_i] + [C_i'] + [C_{i+1}]$ and $E_{iT}' = [E_i'] + [C_i']$ and by the following differential equations:

$$\begin{aligned} \frac{d[Y_i^*]}{dt} &= k_i[C_i] - a_i'[Y_i^*][E_i'] + d_i'[C_i'] - a_{i+1}[Y_{i+1}][Y_i^*] \\ &\quad +(k_{i+1} + d_{i+1})[C_{i+1}] \\ \frac{d[C_i]}{dt} &= a_i[Y_i][Y_{i-1}^*] - (k_i + d_i)[C_i] \\ \frac{d[C_i']}{dt} &= a_i'[Y_i^*][E_i'] - (k_i' + d_i')[C_i'] \end{aligned} \tag{9}$$

with $i = 1, \cdots, n$, with the convention that $[Y_0^*]$ is related to the input stimulus, whereas $[Y_{n+1}] = [C_{n+1}] = 0$.

As described in the main text, we define the parameters: $\epsilon_i = E_{iT}'/Y_{iT}$, $\eta_i = Y_{i-1,T}/Y_{iT}$ and $\mu_i = k_i/k_i'$. We assume that the set of time-scales $\{\epsilon_1 k_1', \epsilon_2 k_2', \cdots, \epsilon_n k_n'\}$ are of the same order and we denote $\epsilon k'$ to be a typical time scale representing this set. We then define a dimensionless time $\tilde{t} = \epsilon k'\, t$ and the time-derivative with respect to $\tilde{t}$ is denoted by a dot, *i.e.* $\dot{x} = dx/d\tilde{t}$. We define also the new variable $[X_i] = [Y_i^*] + [C_{i+1}]$. This definition will let us have, for particular relationships between the parameters of the system, slow and fast variables, respectively. The variables are turned into dimensionless ones in the following way:

$$x_i = \frac{[X_i]}{Y_{iT}}, \quad y_i = \frac{[Y_i]}{Y_{iT}}, \quad c_i = \frac{[C_i]}{Y_{i-1,T}}, \quad c_i' = \frac{[C_i']}{E_{iT}'}, \quad e_i' = \frac{[E_i']}{E_{iT}'}. \tag{10}$$

The system of ODEs can be then written as:

$$\begin{aligned} \dot{x}_i &= \frac{\epsilon_i k_i'}{\epsilon k'} \left(\frac{\mu_i \eta_i}{\epsilon_i} c_i - \frac{a_i' Y_{iT}}{k_i'} (x_i - c_{i+1}) e_i' + \frac{d_i'}{k_i'} c_i' \right) \\ \epsilon\, \dot{c}_i &= \frac{a_i Y_{iT}}{k'} \left(y_i (x_{i-1} - c_i) - K_i c_i \right) \\ \epsilon\, \dot{c}_i' &= \frac{a_i' Y_{iT}}{k'} \left((x_i - c_{i+1}) e_i' - K_i' c_i' \right) \end{aligned} \tag{11}$$

with $i = 1, \cdots, n$, with again the convention that in these equations $c_{n+1} = 0$, and $x_0 = S$ denotes the input stimulus (e.g. some available active enzyme)

normalized by $\eta_1 Y_{1T}$. In fact η_1 could be defined in several ways. As we have chosen to perform numerical simulations with homogeneous $\eta_i = \eta$, $(i = 2, \cdots, n)$, here we choose also $\eta_1 = \eta$. Finally, here the conservation laws look like $x_i + y_i + \eta_i c_i + \epsilon_i c'_i = 1$ and $c'_i + e'_i = 1$.

Now, in the limit where all $\epsilon_i \to 0$, but where $\mu_i \eta_i / \epsilon_i$ are kept of order $O(1)$, we get a fast dynamics for the complexes c_i and c'_i but not for x_i, so that the quasi-steady state approximation can be applied. By imposing that $\dot{c}_i = \dot{c}'_i = 0$, a little calculation gives:

$$c_i = x_{i-1} \frac{y_i}{K_i + y_i}, \qquad c'_i = \frac{(x_i - c_{i+1})}{K'_i + (x_i - c_{i+1})}. \tag{12}$$

In the second equation above (the one for c'_i), c_{i+1} can be replaced using the first equation, *i.e.*, as a function of x_i and y_{i+1}, giving:

$$c'_i = \frac{x_i}{K'_i(1 + y_{i+1}/K_{i+1}) + x_i}.$$

Finally, the substitution of these expressions in Eqs. (11) gives the new model Eq. (6), with $V_i = (k'_i \mu_i \eta_i)/(\epsilon k')$ and $V'_i = (\epsilon_i k'_i)/(\epsilon k')$.

Let us notice that the conservation equation can then be written to the first order in ϵ_i as:

$$x_i + y_i + \eta_i x_{i-1} \frac{y_i}{K_i + y_i} + \epsilon_i \frac{x_i}{K'_i(1 + y_{i+1}/K_{i+1}) + x_i} = 1 \tag{13}$$

where $i = 1, \cdots, n$ and, as usual, $x_0 = S$ and $y_{n+1} = 0$. In principle, the term $O(\epsilon_i)$ could be neglected since it is neglected in the differential equations. In the numerical simulations, however, we have noticed that an easy but significative improvement of the plain truncation of $O(\epsilon_i)$ is achieved by this first order correction.

The model Eq. (6) was derived assuming that the set of time-scales $\{\epsilon_1 k'_1, \epsilon_2 k'_2, \cdots, \epsilon_n k'_n\}$ are of the same order and choosing $\epsilon k'$ to be a typical time-scale representing this set. If this situation does not hold, *i.e.* if those time-scales are not of the same order, but the fastest time-scale is slower than those of the complexes, it is still possible to derive a reduced mechanistic model based on the quasi-steady state approximation. $\epsilon k'$ has to be chosen as the minimum among the values $\{\epsilon_1 k'_1, \epsilon_2 k'_2, \cdots, \epsilon_n k'_n\}$, and then each right hand side has to be multiplied by a factor $(\epsilon_i k'_i)/(\epsilon k')$, being $V_i = \mu_i \eta_i / \epsilon_i$ and $V'_i = 1$. These results were used in the section in the main text that applies the newly derived model to the MAPK pathway.
