# Supplementary material for: A Hidden Feedback in Signaling Cascades Is Revealed
Source: PLoS Comput Biol. 2008 Mar 21;4(3):e1000041. doi: 10.1371/journal.pcbi.1000041 (PMC2265423; doi:10.1371/journal.pcbi.1000041)
Supplement: Text S2 — Available models for signaling cascades using one variable per unit. (0.06 MB PDF) [file pcbi.1000041.s002.pdf]

Text S2
Available models for signaling cascades using one variable per unit

In the main text we have briefly reviewed some approaches followed in the literature to study signaling cascades by means of a single equation per cycle. We add here two more descriptions in the same line and summarize these models with one variable per unit in Table S2.1.

Model	Equations
Weakly activated pathway	$\dot{y}_i^* = \alpha_i y_{i-1}^* - \beta_i y_i^*$<br>[Heinrich et al. (2002)]
Linear rates	$\dot{y}_i^* = \alpha_i y_{i-1}^* y_i - \beta_i y_i^*$<br>with $y_i + y_i^* = 1$<br>[Heinrich et al. (2002)]
GK-like (I)	$\dot{y}_i^* = V_i y_{i-1}^* \frac{y_i}{K_i+y_i} - V_i' \frac{y_i^*}{K_i'+y_i^*}$<br>with $y_i + y_i^* = 1$<br>[Goldbeter and Koshland (1981)]
GK-like (II)	$\dot{y}_i^* = V_i \frac{y_{i-1}^*}{K_{ai}+y_{i-1}^*} \frac{y_i}{K_i+y_i} - V_i' \frac{y_i^*}{K_i'+y_i^*}$<br>with $y_i + y_i^* = 1$<br>[Goldbeter (1991)]<br>[Gonze and Goldbeter (2000)]
Reduced mechanistic	$\dot{x}_i = V_i x_{i-1} \frac{y_i}{K_i+y_i} - V_i' \frac{x_i}{K_i'(1+\frac{y_{i+1}}{K_{i+1}})+x_i}$<br>with $x_i + y_i + \eta_i x_{i-1} \frac{y_i}{K_i+y_i} = 1$<br>and $y_i^* = x_i \frac{K_{i+1}}{K_{i+1}+y_{i+1}}$<br>(this paper)

Table S2. 1: Models for signaling cascades.

In the work of Heinrich et al [Heinrich et al. (2002)], where the linear rates model was studied, the authors also discuss the limiting case called "the weakly activated pathway", where only a small fraction of the targeted protein is activated: $y_i = 1 - y_i^* \simeq 1$. Then, the system given by Eq. (2) in the main text is turned into a system of linear equations, which have been studied extensively in different contexts [Heinrich et al. (2002), Chaves et al. (2004)]:

$$\dot{y}_i^* = \alpha_i y_{i-1}^* - \beta_i y_i^*, \qquad i = 1, \cdots, n, \tag{14}$$

It corresponds to the simplest possible choice, where the kinase and phosphatase reactions are assumed to follow linear rate laws with first-order rate constant, α_i and β_i, respectively.

Besides that one described in the main text, there is another phenomenological extension of the GK model for one cycle that has been proposed in the following form [Goldbeter (1991), Gonze and Goldbeter (2000)]:

$$\dot{y}_i^* = V_i \frac{y_{i-1}^*}{K_{ai} + y_{i-1}^*} \frac{y_i}{K_i + y_i} - V_i' \frac{y_i^*}{K_i' + y_i^*}, \qquad i = 1, \cdots, n. \tag{15}$$

In these equations, the effective maximum rate of the kinase produced at step $i-1$ is proportional to the fraction of active enzyme.

References

[Heinrich et al. (2002)] Heinrich R, Neel BG, Rapoport TA (2002) Mathematical models of protein kinase signal transduction. Mol Cell 9:957-970.

[Goldbeter and Koshland (1981)] Goldbeter A, Koshland DE, Jr (1981) An amplified sensitivity arising from covalent modification in biological systems. Proc Natl Acad Sci USA 78:6840-6844.

[Goldbeter (1991)] Goldbeter A (1991) A minimal cascade model for the mitotic oscillator involving cyclin and cdc2 kinase. Proc Natl Acad Sci USA 88:9107-9111.

[Gonze and Goldbeter (2000)] Gonze D, Goldbeter A (2000) A Model for a Network of Phosphorylation+dephosphorylation Cycles Displaying the Dynamics of Dominoes and Clocks. J Theor Biol 210:167-186.

[Chaves et al. (2004)] Chaves M, Sontag ED, Dinerstein RJ (2004) Optimal length and signal amplification in weakly activated signal transduction cascades. J Phys Chem B 108:15311-15320.
