# Supplementary material for: A Hidden Feedback in Signaling Cascades Is Revealed
Source: PLoS Comput Biol. 2008 Mar 21;4(3):e1000041. doi: 10.1371/journal.pcbi.1000041 (PMC2265423; doi:10.1371/journal.pcbi.1000041)
Supplement: Text S3 — Cascades involving double phosphorylation. (0.23 MB PDF) [file pcbi.1000041.s003.pdf]

Text S3
Cascades involving double phosphorylation

We consider a signaling cascade in which each protein has two phosphorylation sites, as illustrated in Fig. S3.1 . We designate this case as “DP cascade”, to distinguish it from the case where only single phosphorylation occurs (“SP cascade”). Cascades involving both single and double phosphorylation cycles are easily described combining the equations for the SP and DP cascades (as it is the case for the MAPK cascade, which involves SP for the first unit and DP for the second and third ones). The three variables in each cycle in Fig. S3.1 , Y_i, Y_i^*, and Y_i^{**}, represent the three interconvertible forms of the protein, such as the dephosphorylated, singly and doubly phosphorylated forms; the activated form Y_i^{**} acts as a catalyst for the two activation reactions in the next step. In this cascade, the two deactivation reactions in each step share the same phosphatase, denoted by E_i'.

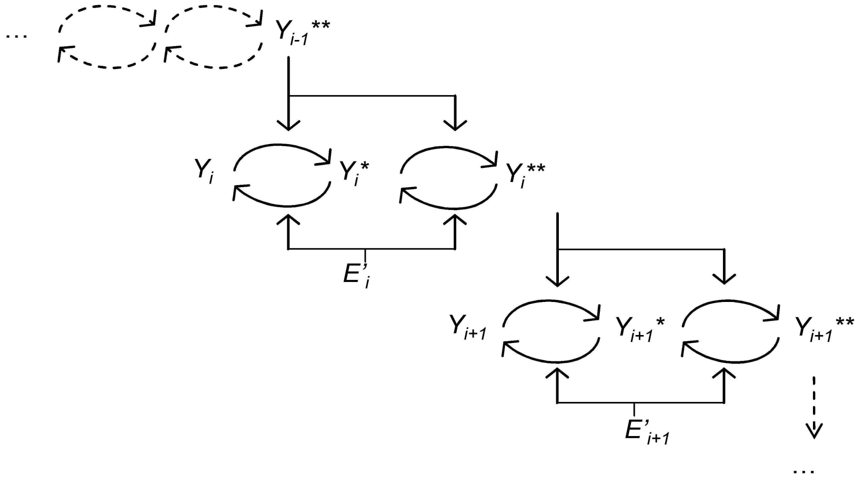

Figure S3. 1: Schematic representation of a cascade of covalent modification cycles, involving double phosphorylation. The ith cycle is composed by three states of the same protein: the inactive, the singly phosphorylated, and the doubly phosphorylated states, labeled Y_i, Y_i^*, and Y_i^{**}, respectively. In each step, the activation is catalyzed by the activated product of the previous step. The deactivation is mediated by another enzyme, E_i'.

Following a similar notation as employed before, the inter-conversion of

the ith protein can be described by the following reactions: :

$$
\begin{aligned}
Y_i + Y_{i-1}^{**} &\underset{d_i}{\overset{a_i}{\rightleftharpoons}} C_i \xrightarrow{k_i} Y_i^* + Y_{i-1}^{**} \\
Y_i^* + Y_{i-1}^{**} &\underset{d_i^*}{\overset{a_i^*}{\rightleftharpoons}} C_i^* \xrightarrow{k_i^*} Y_i^{**} + Y_{i-1}^{**} \\
Y_i^{**} + E_i' &\underset{d_i'}{\overset{a_i'}{\rightleftharpoons}} C_i' \xrightarrow{k_i'} Y_i^* + E_i', \\
Y_i^* + E_i' &\underset{d_i''}{\overset{a_i''}{\rightleftharpoons}} C_i'' \xrightarrow{k_i''} Y_i + E_i',
\end{aligned}
\tag{16}
$$

where C_i, C_i^*, C_i', and C_i'' are intermediate enzyme-substrate complexes. Next, the kinetic equations describing the cascade are written using the law of mass action (the resulting system is the DP mechanistic model) and complemented by the corresponding conservation equations: $Y_{iT} = [Y_i] + [Y_i^*] + [Y_i^{**}] + [C_i] + [C_i^*] + [C_i'] + [C_i''] + [C_{i+1}] + [C_{i+1}^*]$ and $E_{iT}' = [E_i'] + [C_i'] + [C_i'']$.

Four key dimensionless parameters are defined to facilitate the analysis:

$$
\epsilon_i = \frac{E_{iT}'}{Y_{iT}}, \qquad \eta_i = \frac{Y_{i-1,T}}{Y_{iT}}, \qquad \mu_i = \frac{k_i^*}{k_i'}, \qquad \nu_i = \frac{k_i}{k_i''}. \tag{17}
$$

These parameters are analogous to those developed for the SP case. The state of each doubly phosphorylated cycle is found to be described by two variables, y_i and $x_i = y_i^{**} + c_{i+1} + c_{i+1}^*$. As usual, the singular perturbation analysis assumes that the total phosphatase in the cycle is much lower than the total targeted protein, resulting in $\epsilon_i \ll 1$. The other parameters must satisfy:

$$
\mu_i \eta_i \sim \epsilon_i, \qquad \nu_i \eta_i \sim \epsilon_i.
$$

The dynamics of the variables x_i and y_i are described by the differential equations:

$$
\dot{x}_i = V_i x_{i-1} \frac{y_i^*}{K_{\mathit{eff},i}^* + y_i^*} - V_i' \frac{x_i}{K_{\mathit{eff},i}' + x_i}, \tag{18}
$$

$$
\dot{y}_i = U_i' \frac{y_i^*}{K_{\mathit{eff},i}'' + y_i^*} - U_i x_{i-1} \frac{y_i}{K_{\mathit{eff},i} + y_i}, \tag{19}
$$

with the following conservation equation from which y_i^* has to be extracted:

$$
x_i + y_i + y_i^* + \eta_i x_{i-1} \left(\frac{y_i}{K_{\mathit{eff},i} + y_i} + \frac{y_i^*}{K_{\mathit{eff},i}^* + y_i^*} \right) + O(\epsilon_i) = 1. \tag{20}
$$

In the above equations, the parameters are defined by:

$$
V_i = k_i' \mu_i \eta_i, \qquad V_i' = k_i' \epsilon_i, \qquad U_i = k_i'' \nu_i \eta_i, \qquad U_i' = k_i'' \epsilon_i,
$$

and the effective coefficients by:

$$
\begin{aligned}
K_{\mathit{eff},i} &= K_i(1+\frac{y_i^*}{K_i^*}),\\
K^*_{\mathit{eff},i} &= K_i^*(1+\frac{y_i}{K_i}),\\
K'_{\mathit{eff},i} &= K_i'(1+\frac{y_i^*}{K_i''})(1+\frac{y_{i+1}}{K_{i+1}}+\frac{y_{i+1}^*}{K_{i+1}^*}),\\
K''_{\mathit{eff},i} &= K_i''(1+\frac{x_i}{K_i'(1+\frac{y_{i+1}}{K_{i+1}}+\frac{y_{i+1}^*}{K_{i+1}^*})}).
\end{aligned}
$$

Let us notice that Eqs. (18)-(20) in which the effective $K_{\mathit{eff},i}$'s are replaced by the usual Michaelis-Menten constants K_i's, represent the natural extension of the GK-like model to a doubly phosphorylated cascade. This set of extended GK-like equations has been used by several authors in order to study properties of the MAPK pathway [Kholodenko (2000), Angeli et al. (2004)].

The new equations (Eqs. (18)-(20)) are obviously more complicated than the corresponding GK-like equations, or even than the new reduced model presented in this paper for the SP case. In the current study we analyze mostly static properties of Eqs. (18)-(20) and compare them to those of a SP cascade, while a more exhaustive characterization will be presented in a future article.

References

[Kholodenko (2000)] Kholodenko BN (2000) Negative feedback and ultrasensitivity can bring about oscillations in the mitogen-activated protein kinase cascades. Eur J Biochem 267:1583-1588.

[Angeli et al. (2004)] Angeli D, Ferrell JE Jr, Sontag ED (2004) Detection of multistability, bifurcations, and hysteresis in a large class of biological positive-feedback systems. Proc Natl Acad Sci USA 101:1822-1827.
