# Supplementary material for: A Hidden Feedback in Signaling Cascades Is Revealed
Source: PLoS Comput Biol. 2008 Mar 21;4(3):e1000041. doi: 10.1371/journal.pcbi.1000041 (PMC2265423; doi:10.1371/journal.pcbi.1000041)
Supplement: Text S4 — Comparison variables xi and yi*. (0.49 MB PDF) [file pcbi.1000041.s004.pdf]

Text S4
Comparison variables x_i and y_i^*

Fig. S4.1 displays the temporal evolutions corresponding to those in Figs. 3B A_3 and B_3 in the main text, but plotting here variable x_i instead of variable y_i^*. Variable x_i was defined as $x_i = y_i^* + c_{i+1}$, corresponding to the activated protein produced in cycle i and found either in the free form (y_i^*), either being used in the next cycle of the chain (c_{i+1}). Comparing both sets of figures, we observe that the shapes of the curves $x_i(t)$ and $y_i^*(t)$ are qualitatively similar, exhibiting damped oscillations in most cases. Quantitatively, however, variable x_i achieves values of one order of magnitude higher than y_i^*. Therefore these figures corroborate that the level of sequestration is indeed considerable and x_i cannot be used to estimate the available free enzyme created in cycle i of the pathway. Let us remark that for the last unit, the free enzyme is $y_n^* = x_n$. This explains why in our simulations we observe that y_n^* is indeed much larger than the free enzyme available in the previous cycles.

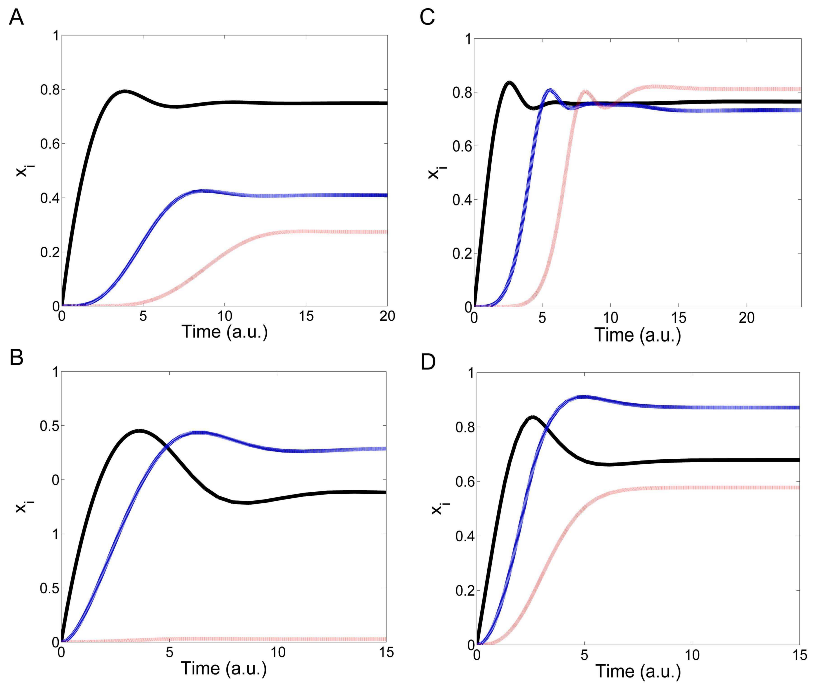

Figure S4. 1: Temporal dynamics for parameters A_3 and B_3 in the main text, with both $n = 10$ and $n = 3$, but plotting variable x_i instead of y_i^*. *a*) A_3 and $n = 10$; *b*) B_3 and $n = 10$; *c*) A_3 and $n = 3$; *d*) B_3 and $n = 3$. $\epsilon = 0.01$, $K = 0.01$, $K' = K/\mu$ and $S = 1$ for all the figures. In every case, time in arbitrary units is plotted in the horizontal axis and the temporal evolution of x_i for only three of the units in the chain is shown: x_1 in filled (black) line, x_4 in dashed (blue) line and x_7 in dotted (red) line, for the cases with $n = 10$. For the cases with $n = 3$, the same line pattern is used for x_1, x_2 and x_3, respectively.
