# Supplementary material for: A Hidden Feedback in Signaling Cascades Is Revealed
Source: PLoS Comput Biol. 2008 Mar 21;4(3):e1000041. doi: 10.1371/journal.pcbi.1000041 (PMC2265423; doi:10.1371/journal.pcbi.1000041)
Supplement: Text S5 — About the amplified “pathway” oscillations. (0.05 MB PDF) [file pcbi.1000041.s005.pdf]

Text S5

About the amplified "pathway" oscillations

The behavior we have described regarding Fig. 3C in the main text can be thought of as a special type of boundary effect. The last unit of the chain, unit n, obeys the same differential equation as the others, but replacing $K'_{eff,n}$ by K'_n, since the last unit is not followed by another one, which consumes or sequesters the activated protein. This result is possibly counterintuitive: even though there is no negative feedback for the last unit, the steady state it achieves is lower than the one that might be expected following the trend of the other units in the chain. Nevertheless, the following is an explanation for this fact. The description for the last unit agrees with the GK-like description, at least within the limit $\eta \simeq \epsilon \ll 1$ where both models share the same conservation law. It was reported in [Goldbeter and Koshland (1984)], studying a single unit with the GK model, that for $V/V' < 1$ and K kept constant, the steady state achieved is smaller, the smaller K' is. Going back to our chain model, V/V' has to be replaced by $x_{i-1}V$. Then, even when $V > 1$ in Fig. 3 and since $x_{n-1} < 1$, the condition $x_n V < 1$ is fulfilled. In addition, we observe that no matter the particular value of K'_{eff}, from its definition, it is clear that it is always greater than K'. As a result, the last unit has a smaller steady state level than the one it would have had with K'_{eff} instead of K'.

The low activation of unit n results in $K'_{eff} >> K'$ for $\dot{x}_{n-1}$. This condition brings the steady-state of unit $n-1$ to a higher level (than that it would have had with $K'_{eff} = K'$), meaning that K'_{eff} is closer to K' for $\dot{x}_{n-2}$. Thus, in the steady state, one can have $x_n < x_{n-1}$ but $x_{n-1} > x_{n-2}$, and so on, for the remaining of the chain.

Even though the effect described is associated with the presence of a boundary, it is far from a mere mathematical artifact. Quite the opposite, cascades in cell signaling are finite rather than infinite chains, so boundary effects are in fact crucial. Furthermore, crosstalk between different pathways could eventually produce an unexpected change in the level of activation of one particular protein, and this effect will propagate not only to the following units in the chain, but also to the preceding ones as well.

We note that a selective amplification of the responses with respect to the amplitude of the first units is achieved, for example, with parameters B_3: the responses of odd indexed units are amplified while responses of units with even index are dampened.

References

[Goldbeter and Koshland (1984)] Goldbeter A, Koshland DE, Jr (1984) Ultrasensitivity in biochemical systems controlled by covalent modifica-

tion. J Biol Chem 259:14441-14447.
