# Supplementary material for: A Hidden Feedback in Signaling Cascades Is Revealed
Source: PLoS Comput Biol. 2008 Mar 21;4(3):e1000041. doi: 10.1371/journal.pcbi.1000041 (PMC2265423; doi:10.1371/journal.pcbi.1000041)
Supplement: Text S6 — More on “reverse” stimulus-response curves. (0.53 MB PDF) [file pcbi.1000041.s006.pdf]

Text S6
More on "reverse" stimulus-response curves

To further explore the possibility of having reverse stimulus-response curves with realistic parameters, we selected another three sets of published parameters, different from the one studied in the main text, but still corresponding to the MAPK signaling pathway [Huang and Ferrel (1996)]. We denote these sets of parameters as "K", "L" and "B/I", respectively ([Kholodenko (2000), Levchenko et al (2000), Bhalla and Iyengar (1999)]), (in honor of the initials of the authors of the papers from where they derive). As depicted in Fig. 7A in the main text, we will consider a 3-unit chain involving only single-phosphorylation. Table S6.1 summarizes the parameters involved in the reduced mechanistic description for each set.

Set-Unit	ϵ	η	μ	K	K'
$K-1$	0.5	1	10	0.1	0.08
$K-2$	0.17	0.33	0.03	0.05	0.05
$K-3$	0.17	1	0.05	0.05	0.05
$L-1$	1	0.67	1	1.67	4
$L-2$	1	1.5	1	0.76	0.45
$L-3$	0.75	0.5	1	0.09	0.25
$B/I-1$	0.11	0.5	0.67	333	78.25
$B/I-2$	0.12	1.11	0.02	0.88	89.94
$B/I-3$	0.0089	0.5	0.15	0.13	0.18

Table S6. 1: Different sets of parameters for the MAPK cascade derived from three different papers [Kholodenko (2000), Levchenko et al (2000), Bhalla and Iyengar (1999)].

Fig. S6.1 *a*) was obtained with the set of parameters K [Kholodenko (2000)]. Since the concentrations of the phosphatases are not specified within this set, we chose them in order to have relatively low values of ϵ_i, as indicated in Table S6.1. The input stimulus to the cascade was taken, in this example to be the concentration of E'_{2T}, the total amount of phosphatase for the middle unit in the cascade. Similarly to what was explained in Fig. 7A, increasing the amount of phosphatase produces a decrease in the response curve y_2^* (filled blue line), as expected. Both the mechanistic and the reduced mechanistic models predict that this perturbation on the second level produces an effect on the first level as well: y_1^* is decreased by the increased demand of y_2. The predictions of the GK-like model are also included in the figure. The inset indicates that a small variation in the control parameter, E'_{2T}, is able to produce a robust 20% change in y_1^*.

Fig. S6.1 *b*) exhibits the results from using the set of parameters named L [Levchenko et al (2000)]. For this particular set, and probably due to

the relatively high values of ϵ_i (see Table S6.1), the agreement between the mechanistic and reduced mechanistic models is not perfect. We then show, for the sake of clarity, only the results of the mechanistic models (dashed lines) and its comparison with the predictions from the GK-like model (dotted lines). The input stimulus to the cascade was taken to be the concentration of Y_{3T}, the total amount of protein for the last unit in the cascade. This perturbation on the third level produces effects on all the levels in the cascade, as indicated in the figure.

Finally, Fig. S6.1 *c*) was obtained with the B/I set in Table S6.1 (this set results from modifying the published set [Bhalla and Iyengar (1999)]). Using the published set of parameters and perturbing only parameters related to concentrations, we could not produce significant reverse stimulus-response curves. We then reduced the concentration of phosphatases one order of magnitude with respect to the published values. With this change, we get a perfect agreement between the mechanistic and reduced mechanistic models and a large effect in level 2 when stimulating level 3. The input stimulus to the cascade was taken to be the concentration of E'_{3T} and the results are qualitatively similar to those shown in Fig. 7A. The inset indicates that a small variation in the stimulus is able to produce a 40% change in y_2^*.

By way of summary, Fig. S6.1 illustrates that the existence of reverse stimulus-response curves is widespread and it could be obtained for very different sets of parameters and different stimulation. Moreover, as indicated in with Fig. S6.1 *b*), it does not require that the system operate in a regime where the reduced mechanistic model is valid. Interestingly, if the parameters in the system are such that there are not reverse responses, a proper manipulation of these parameters can introduce this property in the cascade, as shown in Fig. S6.1 *c*). A more systematic study on how to introduce or remove the reverse response property in a cascade and its applications, is the subject of our current studies, as was mention in regards to Fig. 8B in the main text.

References

[Kholodenko (2000)] Kholodenko BN (2000) Negative feedback and ultrasensitivity can bring about oscillations in the mitogen-activated protein kinase cascades. Eur J Biochem 267:1583-1588.

[Huang and Ferrel (1996)] Huang CY, Ferrell JE, Jr (1996) Ultrasensitivity in the mitogen-activated protein kinase cascade. Proc Natl Acad Sci USA 93:10078-10083.

[Bhalla and Iyengar (1999)] Bhalla US, Iyengar R (1999) Emergent properties of networks of biological signaling pathways. Science 283:381-387.

[Levchenko et al (2000)] Levchenko A, Bruck J, Sternberg PW (2000) Scaffold proteins may biphasically affect the levels of mitogen-activated protein kinase signaling and reduce its threshold properties. Proc Natl Acad Sci USA 97:5818-5823.

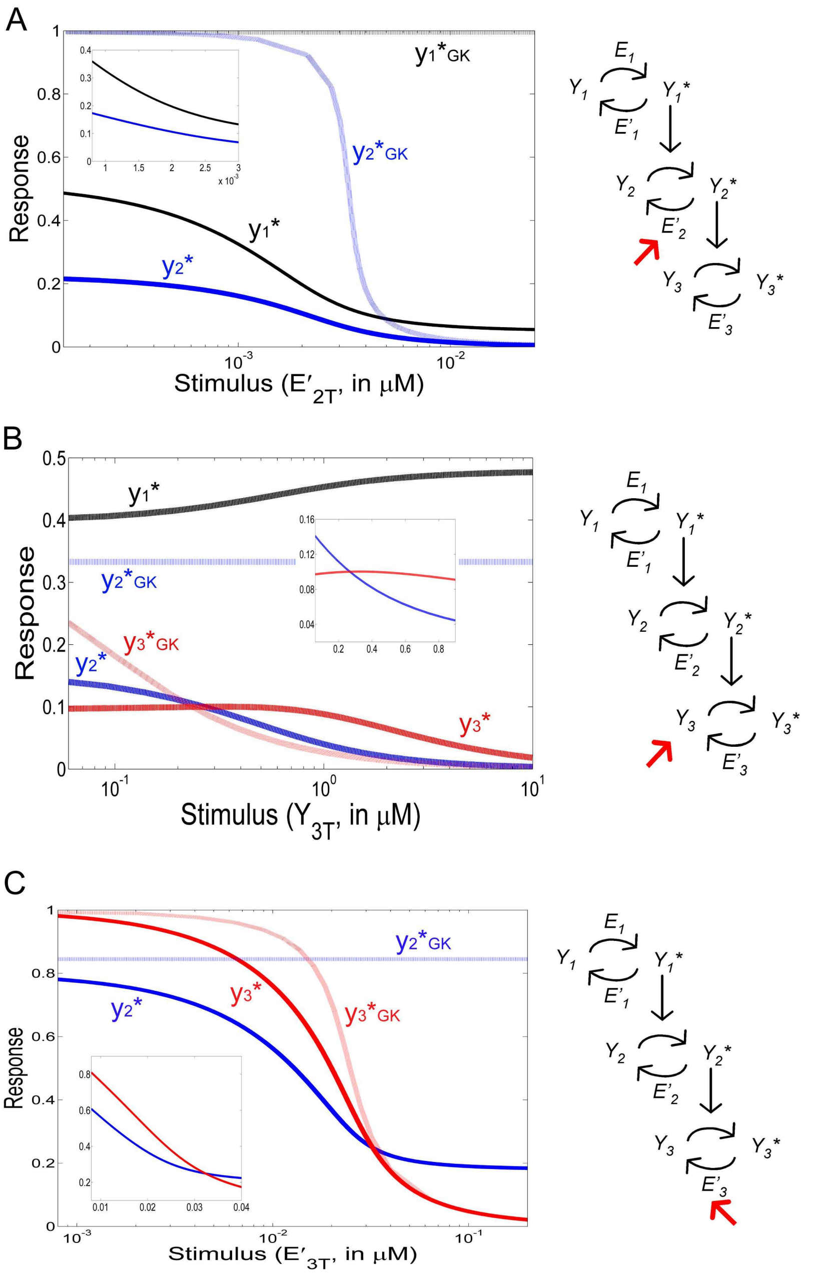

Figure S6. 1: "Reverse" stimulus-response curves for a 3-unit chain involving only single phosphorylation. The parameters in *a*), *b*), and *c*) are those indicated as sets K, L, and B/I, respectively, in Table 3. The input stimulus is given by E'_{2T} in *a*), Y_{3T} in *b*), and E'_{3T} in *c*), and denoted by a red arrow. The responses were obtained with both the mechanistic and the reduced mechanistic descriptions in *a*) and *c*), and with only the mechanistic one in *b*). GK-like model predictions are also included. Insets show enlarged detail of the respective figure.
