# Supplementary material for: A Hidden Feedback in Signaling Cascades Is Revealed
Source: PLoS Comput Biol. 2008 Mar 21;4(3):e1000041. doi: 10.1371/journal.pcbi.1000041 (PMC2265423; doi:10.1371/journal.pcbi.1000041)
Supplement: Text S7 — A variation of the model with an intermediate step in the kinase activation. (0.21 MB PDF) [file pcbi.1000041.s007.pdf]

Text S7
A variation of the model with an intermediate step in the kinase activation

We consider here an alternative scheme for the cascade dynamics in which the activated protein Y_i^* is not directly the kinase for the next reaction. Instead, Y_i^* reacts with a protein P_i giving a complex E_i which will be the activating enzyme for the next reaction, as indicated in Fig. S7.1.

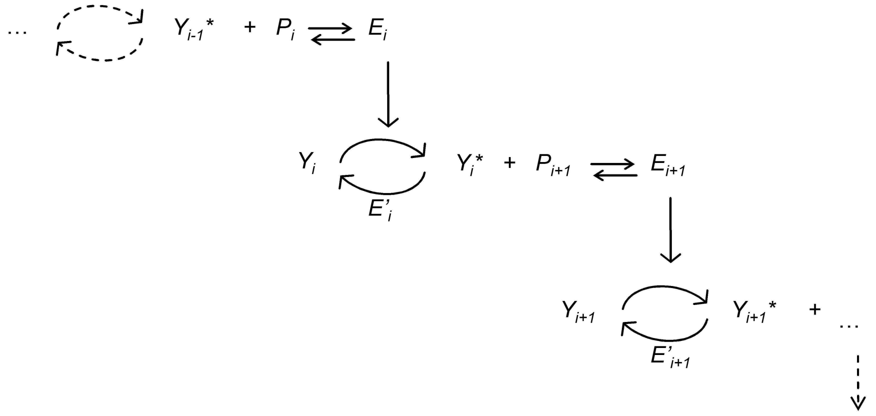

Figure S7. 1: Schematic representation of a cascade of covalent modification cycles. The ith cycle is composed by two states of the same protein, the inactive and the active one, labeled Y_i and Y_i^*, respectively. Y_i^* reacts with protein P_i and the formed complex E_i works as the activating enzyme for the next reaction. The deactivation is performed by another enzyme, E_i'.

In this case, the chemical reactions describing the ith step of the cascade are no longer those in Eq. (1) (main text), but:

$$\begin{aligned} Y_{i-1}^* + P_i \; &\underset{b_i'}{\overset{b_i}{\rightleftharpoons}} \; E_i \\ Y_i + E_i \; &\underset{d_i}{\overset{a_i}{\rightleftharpoons}} \; C_i \xrightarrow{k_i} Y_i^* + E_i \\ Y_i^* + E_i' \; &\underset{d_i'}{\overset{a_i'}{\rightleftharpoons}} \; C_i' \xrightarrow{k_i'} Y_i + E_i' \end{aligned} \tag{21}$$

Unit i in the cascade is now described by four independent variables, since two new variables ($[P_i], [E_i]$) have been added, but there is also one more conservation law: $E_{iT} = [E_i] + [P_i] + [C_i]$. The other two conservation laws become $Y_{iT} = [Y_i] + [Y_i^*] + [C_i] + [C_i'] + [C_{i+1}] + [E_{i+1}]$ and $E_{iT}' = [E_i'] + [C_i']$. Choosing the following independent variables: $[Y_i^*], [E_i], [C_i], [C_i'],$

$(i = 1, \cdots, n)$, and with the law of mass action, the kinetic equations for these variables can be written as follows:

$$\begin{aligned}
\frac{d[Y_i^*]}{dt} &= k_i[C_i] - a_i'[Y_i^*][E_i'] + d_i'[C_i'] - b_{i+1}[Y_i^*][P_{i+1}] + b_{i+1}'[E_{i+1}] \\
\frac{d[E_i]}{dt} &= b_i[Y_{i-1}^*][P_i] - b_i'[E_i] - a_i[Y_i][E_i] + (k_i + d_i)[C_i] \\
\frac{d[C_i]}{dt} &= a_i[Y_i][E_i] - (k_i + d_i)[C_i] \\
\frac{d[C_i']}{dt} &= a_i'[Y_i^*][E_i'] - (k_i' + d_i')[C_i'].
\end{aligned} \tag{22}$$

As in the case without intermediate steps, the goal now is to reduce the number of variables in this system by bringing into play hypothesis that allow one to make the quasi-steady state assumption. It is convenient to work with two new variables defined as $[X_i] = [Y_i^*] + [C_{i+1}] + [E_{i+1}]$ and $[\tilde{E}_i] = [E_i] + [C_i]$. Next, consider the same normalization as in Eqs. (10), except for:

$$c_i = \frac{[C_i]}{E_{iT}}, \qquad \tilde{e}_i = \frac{[\tilde{E}_i]}{E_{iT}}, \qquad p_i = \frac{[P_i]}{E_{iT}}. \tag{23}$$

Now let us define the parameters:

$$\epsilon_i = E_{iT}/Y_{iT}, \qquad \rho_i = E_{iT}'/E_{iT}, \qquad \eta_i = Y_{i-1,T}/Y_{iT}. \tag{24}$$

In what follows, the only assumptions are $\epsilon_i \ll 1$ and the other parameters (ρ_i, η_i) being kept of $O(1)$. This enables us to write $y_i^* = x_i + O(\epsilon)$ and to reduce the dimensionality of the system to only one variable per cycle in the cascade. To show this, let us consider the dimensionless time variable defined as $\tilde{t} = \epsilon k' t$. Then, similarly to the previous derivation, the system of kinetic equations for the new variables is written as:

$$\begin{aligned}
\dot{x}_i &= \frac{\epsilon_i}{\epsilon k'} \left(k_i\, c_i - \rho_i a_i' Y_{iT}\, x_i e_i' + \rho_i d_i' c_i'\right) \\
\epsilon \dot{\tilde{e}}_i &= \frac{b_i \eta_i Y_{iT}}{k'} \left(x_{i-1} p_i - K_{bi} e_i\right) \\
\epsilon\, \dot{c}_i &= \frac{a_i Y_{iT}}{k'} \left(y_i e_i - K_i c_i\right) \\
\epsilon\, \dot{c}_i' &= \frac{a_i' Y_{iT}}{k'} \left(x_i e_i' - K_i' c_i'\right)
\end{aligned} \tag{25}$$

and the conservation laws are $x_i + y_i + \epsilon_i c_i + \epsilon_i \rho_i c_i' = 1$, $\tilde{e}_i + p_i = 1$ and $c_i' + e_i' = 1$. The parameters K_i and K_i' are the Michaelis-Menten constants already defined, and there is a new equilibrium constant $K_{b,i} = b_i'/(b_i Y_{i-1,T})$ which characterizes the activation of the kinase E_i.

Next, the hypothesis $\epsilon \ll 1$ lets one make the standard quasi-steady state assumption, *i.e.*, solving the algebraic equations corresponding to $\dot{\tilde{e}}_i =$

$\dot{c}_i = \dot{c}'_i = 0$. The two latter equations give place to:

$$c_i = \tilde{e}_i \frac{y_i}{K_i + y_i}, \qquad c'_i = \frac{x_i}{K'_i + x_i}. \tag{26}$$

The first equation together with the relation $e_i = \tilde{e}_i - c_i$ leads to $e_i = \tilde{e}_i K_i/(K_i + y_i)$. Combining this last results with the equations $e_i = x_{i-1} p_i / K_{b,i}$ and $p_i = 1 - \tilde{e}_i$, one obtains:

$$\tilde{e}_i = \frac{(K_i + y_i) x_{i-1}}{K_{b,i} K_i + (K_i + y_i) x_{i-1}}.$$

The substitution of this expression in $\dot{x}_i$ finally gives:

$$\dot{x}_i = v_i \frac{y_i}{K_i(1 + K_{b,i}/x_{i-1}) + y_i} - v'_i \frac{x_i}{K'_i + x_i}, \qquad i = 1, \cdots, n, \tag{27}$$

with the simple conservation equation $y_i = 1 - x_i$. The parameters v_i and v'_i are defined as follows: $v_i = (\epsilon_i k_i)/(\epsilon k')$, $v'_i = (\epsilon_i k'_i \rho_i)/(\epsilon k')$.

As a matter of fact, this equation is again different from the GK-type one (Eq. (4)), and no set of assumptions can give the latter as a limit case of the former (except for trivial cases where one recovers in fact the linear system in Eq. (2) in the main text). On the other hand, in the model given by Eq. (27), one sees that there is no longer backward feedback from the each cycle to the previous one.
